# A Distributed Supervisor Architecture for a General Wafer Production System

**DOI:** 10.3390/s23094545

**Published:** 2023-05-07

**Authors:** Fotis N. Koumboulis, Dimitrios G. Fragkoulis, Panteleimon Georgakopoulos

**Affiliations:** 1 Department of Digital Industry Technologies, School of Science, National and Kapodistrian University of Athens, Euripus Campus, 34400 Euboea, Greece; 2 Core Department, National and Kapodistrian University of Athens, Euripus Campus, 34400 Euboea, Greece

**Keywords:** discrete event systems, distributed supervisory control, semiconductor industry

## Abstract

The current trend in the wafer production industry is to expand the production chain with more production stations, more buffers, and robots. The goal of the present paper is to develop a distributed control architecture to face this challenge by controlling wafer industrial units in a general production chain, with a parametric number of production stations, one robot per two stations where each robot serves its two adjacent production stations, and one additional robot serving a parametric number of stations. The control architecture is analyzed for individual control units, one per robot, monitoring appropriate event signals from the control units of the adjacent robots. Each control unit is further analyzed to individual supervisors. In the present paper, a modular parametric discrete event model with respect to the number of production stations, the number of buffers, and the number of robotic manipulators is developed. A set of specifications for the total system is proposed in the form of rules. The specifications are translated and decomposed to a set of local regular languages for each robotic manipulator. The distributed supervisory control architecture is developed based on the local regular languages, where a set of local supervisors are designed for each robotic manipulator. The desired performance of the total manufacturing system, the realizability, and the nonblocking property of the proposed architecture is guaranteed. Finally, implementation issues are tackled, and the complexity of the distributed architecture is determined in a parametric formula. Overall, the contribution of the present paper is the development of a parametric model of the wafer manufacturing systems and the development of a parametric distributed supervisory control architecture. The present results provide a ready-to-hand solution for the continuously expanding wafer production industry.

## 1. Introduction

One of the main goals of Industry 4.0 is to improve existing systems and processes via the automatization of manufacturing processes and the interoperability of corresponding applications using cyber-physical systems and the Internet of Things (IoT). The adaptation of these tools in many systems’ processes, including installed robotic manipulators, and the direction for more flexible and easily expandable manufacturing systems increase the need for smarter and more efficient control systems. One of the manufacturing sectors into which these tools are introduced at many different stages of the manufacturing process is the semiconductor manufacturing industry [1].

In semiconductor industry, wafer processing units have an important role. The wafer processing unit (see [2,3,4,5,6,7,8,9]) is a manufacturing unit consisting of a set of integrated production stations (process chambers), a set of one-slot buffers, one loading dock (input) of the raw products, one loading dock (output) of the manufactured products, and a set of robotic manipulators that transfer the products between the stations, the buffers, and the input and output docks. For every production station, there is one robotic manipulator dedicated to serve the production station. The production station and the robotic manipulator constitute an individual manufacturing unit, called a cluster tool. The first studies of wafer manufacturing units (see [2,3,4,5,6,7]) have been limited to single cluster tool systems up to four cluster tools systems. The systems that have more than one cluster tool are called multicluster tool systems. The current trend in the wafer industry is to increase the number of cluster tools participating in the process (see [8,9]), or/and to increase the number of production stations in each cluster tool (see [10,11]). 

For the case of multicluster tool systems, with a parametric number of production stations in each cluster, the number of studies towards synchronization of the cluster tool and scheduling analysis of the manufacturing unit is increasing (indicatively, see [10,11,12,13,14]). The goal of these studies is to increase the production speed and optimize the job scheduling of the manufacturing unit. According to [10], multicluster tool systems can be viewed either from the point of view of the robots or from the point of view of the wafer product flow. In the first case, the production times are considered to be neglectable. Therefore, the study is focused on the supervision of the pick-and-place tasks of the robotic manipulators. In the second case, the processing time is considered to be non-neglectable, while the pick-and-place times are considered to be neglectable. Therefore, the study focuses on the flow of wafers and the processing timing of the wafers in the production sections. In the present paper, the study focuses on the supervision of the robotic manipulators, and the production time is considered to be zero.

The architecture of a cluster tool in the semiconductor industry has been presented in [2], where linear wafer processing has been introduced. In [2], the system’s analysis focuses on the time needed for the manufacturing of the wafers to be completed. In [3,4], a wafer processing unit, consisting of nine production stations, three buffers, and four robotic manipulators, is studied. In [3], the wafer processing units are modelled using finite deterministic automata. The automaton of the stations and the buffers has a deadlock. In [3], a set of local supervisors is proposed to avoid the deadlock and coordinate the transfer of the wafers from one production unit to another. In [4], the models developed in [3] have been used and a set of supervisors is proposed to avoid deadlock and coordinate the overall manufacturing unit. In [5,6], a wafer manufacturing unit with two production stations, one buffer, and two robotic manipulators is studied. Furthermore, a set of local supervisors is proposed for the coordination of the unit. In [7], an extended version of the wafer manufacturing unit, consisting of eleven production stations, four buffers, and five robotic manipulators, is studied. In [7], the models of the stations and the buffers are not used and the supervisor design is based on the models of the robotic manipulators. The models of the manipulators can be viewed as controlled versions of the models in [3,4]. In [7], about 70 local supervisors are derived to prevent overflow of the stations and buffers as well as unnecessary use of manipulators. In [8,9], a wafer production line, with a parametric number of robots, is studied. Each robot serves two production stations, except for the last robot, which serves three production stations. The design specifications in [8,9] guarantee nonblocking through a distributed supervisory control scheme including a set of four two-state supervisors for each robotic manipulator and a global supervisor counting the wafers. In [8,9], the design of supervisors is based on abstractions of the automata models of the process in [3]. Both works focus on the time needed for the completion of manufacturing.

In the present paper, a modular discrete event model of a wafer manufacturing unit, being parametric with respect to the number of production stations, the number of buffers, and the number of robotic manipulators, is developed using discrete event systems (DES) and especially the Ramadge–Wonham (RW) framework (see [15,16]). The configuration of the present wafer manufacturing unit consists of a chain of a parametric number of robots, where each robot serves two production stations, and two buffers. The last manipulator serves another parametric number of production stations. The model covers the models in [8,9] as special cases. This general model is the first contribution of the paper. The desired specification is expressed in the form of five rules. The specifications are translated and decomposed to appropriate sets of local regular languages, one per robotic manipulator. A set of parametric supervisors in the form of finite deterministic automata and in the RW framework is developed. Furthermore, a distributed supervisory control architecture is developed. For distributed supervisory control and its properties, see [15]. The architecture consists of appropriate control units, one per robotic manipulator, monitoring appropriate event signals of the adjacent control units. The development of the present distributed supervisory control, with parametric supervisors, is the second contribution of the paper. The desired performance of the controlled total manufacturing system, the realizability of the control structure (for the notion of realizability see [17]), and the nonblocking property (see [15]) of the proposed architecture are guaranteed. Finally, implementation issues are tackled, and the complexity of the distributed architecture is determined in a parametric formula. The final contribution of the paper is the development of a clear and sufficient method to face possible extensions of the number of production stations and robotic manipulators in the wafer industry. The motivations of the present research were (a) the introduction of a generic modelling for wafer manufacturing in the semiconductor industry, and (b) the cover of the current industrial trends by the extension of the number of the production stations and the number of the serving robotic manipulators. An additional significant motivation was to provide a generic distributed supervisory control architecture, with respect to the system’s devices, that will be able to adopt a system’s specifications to any possible extensions of the system. 

This study is structured as follows: In Section 2, the DES models of the components of the parametric wafer manufacturing system are presented. In Section 3, the desired behavior of the manufacturing process is expressed in the form of a set of desired regular languages. In Section 4, the distributed supervisory control architecture is analyzed. In Section 5, the supervisor automata realizing the desired regular languages are presented. In Section 6, the required system properties of the controlled automaton of the wafer manufacturing system are investigated. In Section 7, simulation results of the controlled automaton regarding a practical sequence of operating commands are presented. In Section 8, the total complexity of the proposed distributed supervisory scheme is calculated. Finally, in Section 9, implementation aspects of the proposed scheme are presented, and the implementation of the proposed supervisors in the ladder diagram (LD) framework are developed.

## 2. Modelling of the Manufacturing Process

### 2.1. Parametric Notation and Configuration of the Process 

As already mentioned, the wafer manufacturing system consists of production stations, buffers, two loading docks (one for the raw product and one for the manufactured products), and a set of robotic manipulators dedicated to the product transfer between the stations and the two docks. Here, the process is considered in parametric form. The number of robotic manipulators is equal to n+1, where $n\in {\mathbb{Z}}_{+}$ and ${\mathbb{Z}}_{+}$ comprise the set of positive integers. Each robotic manipulator is indexed by the integer i, where

(1a) i∈{1,…,n+1}

The i-th robotic manipulator is denoted by $R_{i}$. It is important to mention that if i∈{1,…,n}, then every $R_{i}$ serves two production stations, configured as a couple in the production line, while if i=n+1, then $R_{i}$ serves a parametric number of $m\in {\mathbb{Z}}_{+}$ production stations. The total number of production stations is equal to 2n+m. Furthermore, each production station is indexed by the integers i and j, where

(1b) $$j\in J(i);\;J(i)=\left\{\begin{array}{cc} \{1,2\}, & \mathrm{if}\;i\in \{1,\ldots ,n\}\\ \{1,2,\ldots ,m\}, & \mathrm{if}\;i=n+1 \end{array}\right.$$

According to (1b), the set J(i), namely the range of j, depends upon i. The index i is the index of the robot manipulator serving the (i,j) station. The index j is the index of the production station served by the i-th robot. So, each production station is denoted by $C_{i,j}$. The number of buffers is equal to n. Each buffer is indexed by the integer ν, where

(1c) ν∈{1,…,n}

The symbol $B_{\nu }$ represents the ν-th buffer. The first loading dock is the input dock of the process and is represented by $L_{in}$. The second loading dock is the output dock of the process and is represented by $L_{out}$. 

To illustrate the above integer definitions, an example is presented. Let n=3 and m=3. Therefore it holds that J(1)={1,2}, J(2)={1,2}, J(3)={1,2}, and J(4)={1,2,3}. Thus, there are four robotic manipulators, denoted by $R_{1}$, $R_{2}$, $R_{3}$, and $R_{4}$, and three buffers, denoted by $B_{1}$, $B_{2}$, and $B_{3}$. The number of production stations is equal to nine, and they are denoted by $C_{1,1}$, $C_{1,2}$, $C_{2,1}$, $C_{2,2}$, $C_{3,1}$, $C_{3,2}$, $C_{4,1}$, $C_{4,2}$, and $C_{4,3}$.

**Figure 1:**
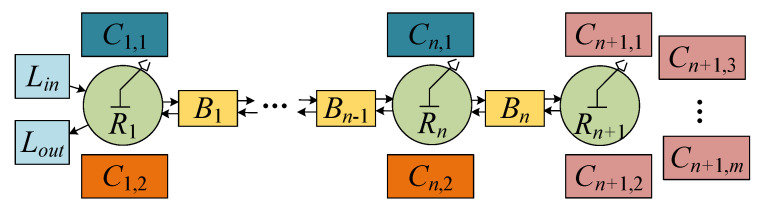
Drawing of the configuration of the wafer manufacturing system.

In Figure 1, a simplified drawing of the configuration of the components of the wafer production process is presented. The configuration of the array of the production stations as well as the array of the robotic manipulators and the array of the buffers allows appropriate transfer of products. In particular, this process enables the following:

(i) $R_{1}$ to transfer products from $L_{in}$ to $C_{1,1}$, from $C_{1,1}$ and $C_{1,2}$ to $B_{1}$, and from $C_{1,2}$ to $L_{out}$,
(ii) $R_{i}$, where i∈{2,…,n}, to transfer products from $C_{i,1}$ and $C_{i,2}$ to $B_{i}$ and $B_{i-1}$, and vice versa, i.e., from $B_{i}$ and $B_{i-1}$ to $C_{i,1}$ and $C_{i,2}$,
(iii) $R_{n+1}$ to transfer products from $C_{n+1,1}$,…,$C_{n+1,m}$ to $B_{n}$ and vice versa.

In the following subsections, the physical entities $R_{i}$, $C_{i,j}$, $B_{\nu }$, $L_{in}$, and $L_{out}$, composing the production unit, will be modelled by mathematical entities in DES forms. In particular, the DES model of $R_{i}$ is denoted by GiR, the DES model of $C_{i,j}$ is denoted by Gi,j, the DES model of $B_{\nu }$ is denoted by Gi, the DES model of $L_{in}$ is denoted by $G_{I}$, and the DES model of $L_{out}$ is denoted by $G_{O}$.

### 2.2. The Models of the Production Stations

The model of the production station $C_{i,j}$ is described by the 6-tuple

(2) Gi,j=(ℚi,j,Ei,j,fi,j,ℍi,j,xi,j0,ℚi,jm)

For the description of finite deterministic automata in the form of a 6-tuple, see [17,18,19,20,21]. The set of the states of the automaton is

(3) ℚi,j={qi,j1,qi,j2,qi,j3,qi,j4}

The state qi,j1 describes the case where there is no product at $C_{i,j}$ and the station is in idle mode (standby). The state qi,j2 describes the case where there is a product at the station, dropped by $R_{i}$. The state qi,j3 describes the case where the processing of the product at the station has been completed and the processed product has not yet been picked up by $R_{i}$. The state qi,j4 describes two malfunction cases. The first is the case where $R_{i}$ tried to pick a product when there was no processed product at the station. The second is the case where $R_{i}$ dropped a product to the station when there was a product at the station. Clearly, qi,j4 describes a functional faulty situation. The initial state is xi,j0=qi,j1. The set of the marked states of the automaton is ℚi,jm={qi,j1,qi,j2,qi,j3}. 

The alphabet of the automaton is

(4) Ei,j={ei,jP,ei,jD,ei,ju}

The event ei,jP is the command to $R_{i}$ to pick a product from the station. The event ei,jD is the command to $R_{i}$ to drop a product at the station. The event ei,ju takes place when the processing of the product has been completed. The set of the controllable events of the automaton is Ei,jc={ei,jD,ei,jP}. Hence, the set of the uncontrollable events is Ei,juc={ei,ju}. 

The values of the transition function of the automaton are:

fi,j(qi,j1,ei,jD)=qi,j2, fi,j(qi,j2,ei,ju)=qi,j3, fi,j(qi,j3,ei,jP)=qi,j1, fi,j(qi,j1,ei,jP)=qi,j4, fi,j(qi,j2,ei,jD)=qi,j4, fi,j(qi,j2,ei,jP)=qi,j4, fi,j(qi,j3,ei,jD)=qi,j4.

The active event sets of the automaton are:

ℍi,j(qi,j1)={ei,jD,ei,jP}, ℍi,j(qi,j2)={ei,jD,ei,jP,ei,ju}, ℍi,j(qi,j3)={ei,jD,ei,jP}, ℍi,j(qi,j4)=∅

The closed behavior of the automaton is

(5) L(Gi,j)=(ei,jDei,juei,jP)*ei,jP+ei,jD(ei,jD+ei,jP+ei,juei,jD)¯

and the marked behavior of the automaton is

(6) Lm(Gi,j)=(ei,jDei,juei,jP)*¯

where the symbol “${}^{*}$” denotes the Kleene star of a language. Regarding the Kleene star, see [7,16]. Regarding the definition and properties L(·) and $L_{m}(\cdot )$, namely the closed and the marked behavior of the argument automaton, see [7]. The automaton Gi,j in (2) is a blocking automaton, as L(Gi,j)≠Lm(Gi,j)¯. 

**Figure 2:**
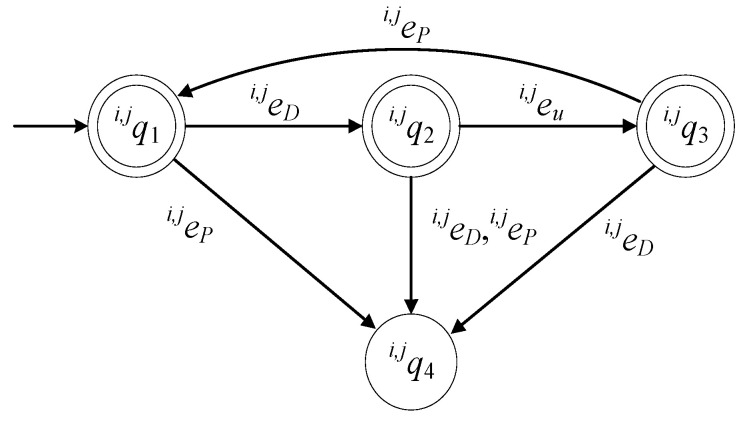
State diagram of the model of $C_{i,j}$.

In Figure 2, the state diagram of Gi,j is presented. The state diagram of Gi,j is the same as the state diagram of the model of the production station proposed in [4].

### 2.3. The Models of the Buffers

The model of $B_{\nu }$ is described by the 6-tuple

(7) Gν=(ℚν,Eν, fν,ℍν,xν0,ℚνm)

The set of the states of the automaton is

(8) ℚν={qν1,qν2,qν3,qν4}

The state qν1 describes the case where the buffer is empty. The state qν2 describes the case where there is a product at the buffer, dropped by $R_{\nu }$. The state qν3 describes the case where there is a product at the buffer, dropped by $R_{\nu +1}$. The state qν4 describes two faulty cases. The first is the case where $R_{\nu }$ (or $R_{\nu +1}$) tried to pick a product when the buffer was empty. The second is the case where $R_{\nu }$ (or $R_{\nu +1}$) dropped a product when the buffer was full. The initial state of the automaton is xν0=qν1. The set of the marked states of the automaton is ℚνm={qν1,qν2,qν3}.

The alphabet of the automaton is

(9) Eν={eν,νBP,eν,νBD,eν+1,νBP,eν+1,νBD}

The event eν,νBP is the command to $R_{\nu }$ to pick a product from $B_{\nu }$. The event eν,νBD is the command to $R_{\nu }$ to drop a product to $B_{\nu }$. The event eν+1,νBP is the command to $R_{\nu +1}$ to pick a product from $B_{\nu }$. The event eν+1,νBD is the command to $R_{\nu +1}$ to drop a product to $B_{\nu }$. The set of the controllable events of the automaton is Eνc=Eν. Hence, the set of the uncontrollable events is Eνuc=∅.

The values of the transition functions of the automaton are:

fν(qν1,eν,νBD)=qν2, fν(qν1,eν+1,νBD)=qν3, fν(qν2,eν+1,νBP)=qν1, fν(qν3,eν,νBP)=qν1, fν(qν1,eν+1,νBP)=qν4, fν(qν1,eν,νBP)=qν4, fν(qν2,eν,νBP)=qν4, fν(qν2,eν,νBD)=qν4, fν(qν2,eν+1,νBD)=qν4, fν(qν3,eν,νBD)=qν4, fν(qν3,eν+1,νBP)=qν4, fν(qν3,eν+1,νBD)=qν4

The active event sets of the automaton are

ℍν(qν1)=ℍν(qν2)=ℍν(qν3)=Eν, ℍν(qν4)=∅

The closed behavior of the automaton is

(10) L(Gν)=(eν,νBDeν+1,νBP+eν+1,νBDeν,νBP)*eν,νBP+eν+1,νBP+eν,νBD(eν,νBD+eν,νBP+eν+1,νBD)+eν+1,νBD(eν,νBD+eν+1,νBD+eν+1,νBP)¯

and the marked behavior of the automaton is

(11) Lm(Gν)=(eν,νBDeν+1,νBP+eν+1,νBDeν,νBP)*¯

The automaton Gν in (7) is a blocking automaton, as L(Gν)≠Lm(Gν)¯. 

**Figure 3:**
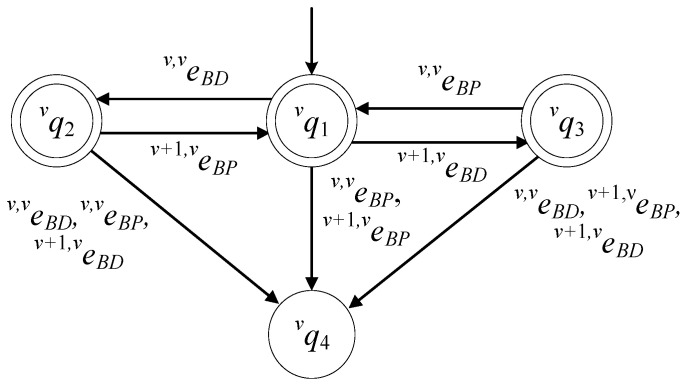
State diagram of the model of $B_{\nu }$.

In Figure 3, the state diagram of Gν is presented. The state diagram of Gν is the same as the model of the buffer proposed in [4].

### 2.4. The Models of the Loading Docks

The model of $L_{in}$ (input loading dock) is described by the 6-tuple

(12) $$G_{I}=({\mathbb{Q}}_{I},E_{I},f_{I},{ℍ}_{I},x_{I,0},{\mathbb{Q}}_{I,m})$$

The set of the states of the automaton is ${\mathbb{Q}}_{I}=\{q_{I,1}\}$. The state $q_{I,1}$ is the only state of the automaton. The initial state of the automaton is $x_{I,0}=q_{I,1}$. The set of the marked states is ${\mathbb{Q}}_{I,m}=\{q_{I,1}\}$.

The alphabet of the automaton is $E_{I}=\{e_{I}\}$. The event $e_{I}$ is the command to $R_{1}$ to pick a product from $L_{in}$. The set of the controllable events is $E_{I,c}=\{e_{I}\}$. Hence, the set of the uncontrollable events is $E_{I,uc}=\emptyset$. The transition function is $f_{I}(q_{I,1},e_{I})=q_{I,1}$. The active event set is ${ℍ}_{I}(q_{I,1})=\{e_{I}\}$. 

**Figure 4:**
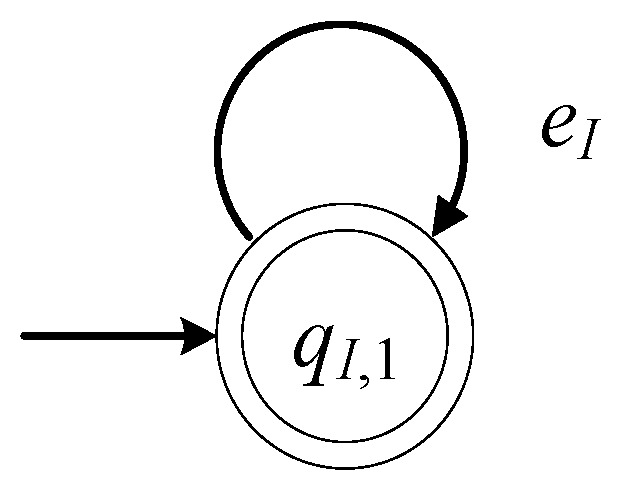
State diagram of the model of $L_{in}$.

The closed behavior of the automaton is equal to the marked behavior of the automaton, i.e., $L(G_{I})=L_{m}(G_{I})=e_{I}^{*}$. Thus, $G_{I}$ in (12) is a nonblocking automaton. In Figure 4, the state diagram of $G_{I}$ is presented.

The model of $L_{out}$ (output loading dock) is described by the 6-tuple

(13) $$G_{O}=({\mathbb{Q}}_{O},E_{O},f_{O},{ℍ}_{O},x_{O,0},{\mathbb{Q}}_{O,m})$$

The set of the states of the automaton is ${\mathbb{Q}}_{O}=\{q_{O,1}\}$. The state $q_{O,1}$ is the only state of the automaton. The initial state of the automaton is $x_{O,0}=q_{O,1}$. The set of the marked states of the automaton is ${\mathbb{Q}}_{O,m}=\{q_{O,1}\}$.

The alphabet of the automaton is $E_{O}=\{e_{O}\}$. The event $e_{O}$ is the command to $R_{1}$ to drop a product to $L_{out}$. The set of the controllable events is $E_{O,c}=\{e_{O}\}$. Hence, the set of the uncontrollable events is $E_{O,uc}=\emptyset$. The transition function of the automaton is $f_{O}(q_{O,1},e_{O})=q_{O,1}$. The active events set is ${ℍ}_{O}(q_{O,1})=\{e_{O}\}$. 

**Figure 5:**
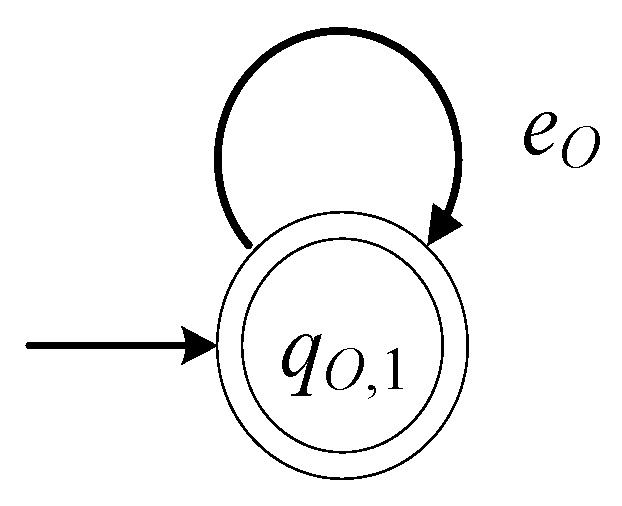
State diagram of the model of $L_{out}$.

The closed behavior of the automaton is equal to the marked behavior of the automaton, i.e., $L(G_{O})=L_{m}(G_{O})=e_{O}^{*}$. Thus, $G_{O}$ in (13) is a nonblocking automaton. In Figure 5, the state diagram of $G_{O}$ is presented.

The state diagrams of each of the two loading docks, in Figure 4 and Figure 5, are the same with the respective models in [4].

### 2.5. The Models of the Robotic Manipulators

The model of $R_{1}$ is described by the 6-tuple

(14) G1R=(ℚ1R,E1R, f1R,ℍ1R,x1R,0,ℚ1R,m)

The set of the states of the automaton is ℚ1R={q1R,1,q1R,2}. The state q1R,1 describes the case where the gripper of $R_{1}$ is empty. The state q1R,2 describes the case where a product is gripped by $R_{1}$. The initial state of the automaton is x1R,0=q1R,1. The set of the marked states is ℚ1R,m={q1R,1}.

The alphabet of the automaton is

(15) E1R={eI,eO,e1,1P,e1,1D,e1,2P,e1,2D,e1,1BP,e1,1BD}

The events of the above alphabet have been defined in the previous subsections. It is important to mention that all events of E1R in (15) are controllable.

The values of the transition function of the automaton are

f1R(q1R,1,e)=q1R,2, ∀e∈{eI,e1,1P,e1,2P,e1,1BP}, f1R(q1R,2,e)=q1R,1, ∀e∈{eO,e1,1D,e1,2D,e1,1BD}

The active event sets of the automaton are

ℍ1R(q1R,1)={eI,e1,1P,e1,2P,e1,1BP}, ℍ1R(q1R,2)={eO,e1,1D,e1,2D,e1,1BD}

The closed behavior of the automaton is

(16) L(G1R)=(eI+e1,1P+e1,2P+e1,1BP)(eO+e1,1D+e1,2D+e1,1BD)*¯

and the marked behavior of the automaton is

(17) Lm(G1R)=(eI+e1,1P+e1,2P+e1,1BP)(eO+e1,1D+e1,2D+e1,1BD)*

Thus, G1R in (14) is a nonblocking automaton. 

**Figure 6:**
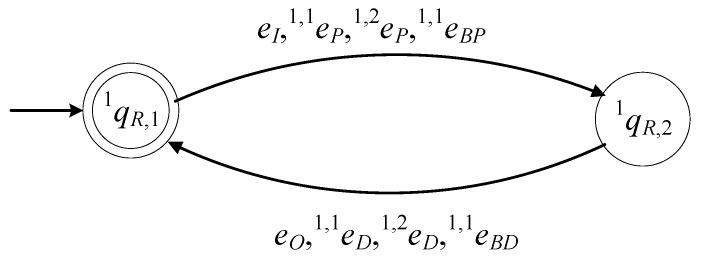
State diagram of the model of $R_{1}$.

In Figure 6, the state diagram of G1R is presented.

The model of $R_{i}$ where i={2,…,n} is described by the 6-tuple

(18) GiR=(ℚiR,EiR, fiR,ℍiR,xiR,0,ℚiR,m)

The set of the states of the automaton is

(19) ℚiR={qiR,1,qiR,2}

The state qiR,1 describes the case where the gripper of $R_{i}$ is empty. The state qiR,2 describes the case where a product is gripped by $R_{i}$. The initial state of the automaton is xiR,0=qiR,1. The set of the marked states of the automaton is ℚiR,m={qiR,1}.

The alphabet of the automaton is

(20) EiR={ei,1P,ei,1D,ei,2P,ei,2D,ei,i−1BP,ei,i−1BD,ei,iBP,ei,iBD}

The events of the above alphabet have been defined in the previous subsections. It is important to mention that all events of EiR in (20) are controllable. 

The values of the transition function of the automaton are

fiR(qiR,1,e)=qiR,2, ∀e∈{ei,1P,ei,2P,ei,i−1BP,ei,iBP}, fiR(qiR,2,e)=qiR,1, ∀e∈{ei,1D,ei,2D,ei,i−1BD,ei,iBD}

The active event sets of the automaton are

ℍiR(qiR,1)={ei,1P,ei,2P,ei,i−1BP,ei,iBP}, ℍiR(qiR,2)={ei,1D,ei,2D,ei,i−1BD,ei,iBD}

The closed behavior of the automaton is

(21) L(GiR)=(ei,1P+ei,2P+ei,i−1BP+ei,iBP)(ei,1D+ei,2D+ei,i−1BD+ei,iBD)*¯

and the marked behavior of the automaton is

(22) Lm(GiR)=(ei,1P+ei,2P+ei,i−1BP+ei,iBP)(ei,1D+ei,2D+ei,i−1BD+ei,iBD)*

Hence, GiR in (18) is a nonblocking automaton. 

**Figure 7:**
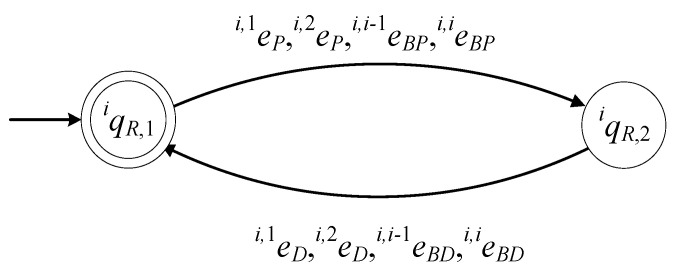
State diagram of the model of $R_{i}$.

In Figure 7, the state diagram of GiR is presented.

The model of $R_{n+1}$ is described by the 6-tuple

(23) Gn+1R=(ℚn+1R,En+1R,fn+1R,ℍn+1R,xn+1R,0,ℚn+1R,m)

The set of the states is ℚn+1R={qn+1R,1,qn+1R,2}. The state qn+1R,1 describes the case where the gripper of $R_{n+1}$ is empty. The state qn+1R,2 describes the case where a product is gripped by $R_{n+1}$. The initial state of the automaton is xn+1R,0=qn+1R,1. The set of the marked states of the automaton is ℚn+1R,m={qn+1R,1}.

The alphabet of the automaton is

(24) En+1R=∪j=1m{en+1,jP,en+1,jD}∪{en+1,nBP,en+1,nBD}

The events of the above alphabet have been defined in the previous subsections. It is important to mention that all events of En+1R in (24) are controllable. 

The values of the transition function of the automaton are

fn+1R(qn+1R,1,e)=qn+1R,2, ∀e∈∪j=1m{en+1,jP}∪{en+1,nBP}, fn+1R(qn+1R,2,e)=qn+1R,1, ∀e∈∪j=1m{en+1,jD}∪{en+1,nBD}

The active event sets of the automaton are

ℍn+1R(qn+1R,1)=∪j=1m{en+1,jP}∪{en+1,nBP}, ℍn+1R(qn+1R,2)=∪j=1m{en+1,jD}∪{en+1,nBD}

The closed behavior of the automaton is

(25) L(Gn+1R)=+j=1men+1,jP+en+1,nBP+j=1men+1,jD+en+1,nBD*¯

and the marked behavior of the automaton is

(26) Lm(Gn+1R)=+j=1men+1,jP+en+1,nBP+j=1men+1,jD+en+1,nBD*

Hence, Gn+1R in (23) is a nonblocking automaton. 

**Figure 8:**
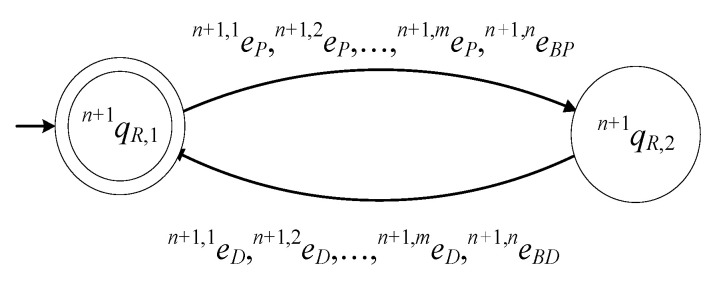
State diagram of the model of $R_{n+1}$.

In Figure 8, the state diagram of Gn+1R is presented.

For the case where n=3 and m=3, the models of the robotic manipulators, presented in this subsection, are reduced to those presented by respective state diagrams in [4].

## 3. Desired Behavior

### 3.1. The Rules of the Desired Behavior 

**Figure 9:**
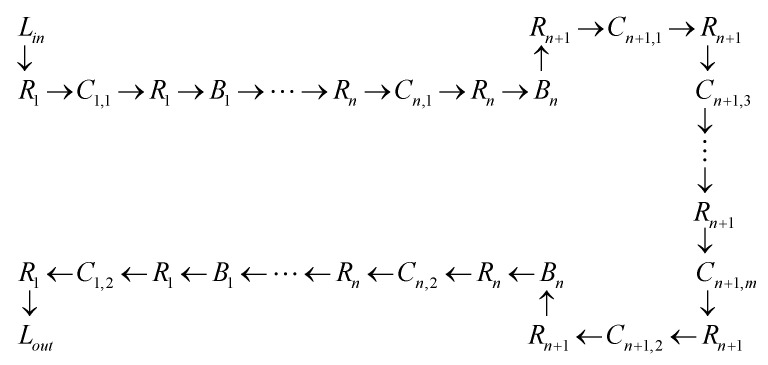
Desired production sequence.

The desired behavior of the wafer manufacturing system can be described in the form of rules as follows:

- Only if a station (or a buffer) is empty will the respective serving robotic manipulator drop a product to the station (or the buffer).
- Only if a station has a manufactured product (or a buffer has a product) will the respective serving robotic manipulator pick a product from the station (or the buffer).
- The desired production sequence, namely the sequence of the placements of a wafer, is presented in the transportation diagram in Figure 9.
- Once a wafer is picked up by $R_{i}$, where i∈{1,…,n}, if $C_{i+1,1}$ is full, then the transportation of the wafer to $B_{i}$ may cause blocking. Hence, a reasonable requirement to avoid blocking is to guarantee an empty slot in $C_{i+1,1}$ before $R_{i}$ initiates the wafer transportation to $B_{i}$.
- Once a wafer is picked up by $R_{i}$, where i∈{2,…,n+1}, if $C_{i-1,2}$ is full, then the transportation of the wafer to $B_{i-1}$ may cause blocking. Hence, a reasonable requirement to avoid blocking is to guarantee an empty slot in $C_{i-1,2}$ before $R_{i}$ initiates the wafer transportation to $B_{i-1}$.

The first rule is a modification of the respective rule in [7]. It is noted that the uncontrollable events, signaling completion of the production process, have not been considered in the model of [7]. The second rule is more restrictive than the respective rule in [7], in the sense that it does not allow the respective robotic manipulator to pick a product if the destination buffer is full. According to the respective rule in [7], the robotic manipulator is not allowed to drop a product to the buffer only if the buffer is full. The third rule is introduced here to guarantee the serial structure of the wafer manufacturing system. This rule is in accordance with the production sequence presented in [7] for the case where n=4 and m=3. The fourth rule has been introduced first here. The aim of the fourth rule is to avoid blocking when transporting products from the machines $C_{i,1}$ to the respective buffer. The fifth rule was first introduced in [7].

### 3.2. The Desired Behavior in the Form of Regular Languages 

For $C_{i,j}$, where i∈{1,…,n+1} and j∈J(i), which is given in (1), the first and second rule can be expressed in the form of the following regular languages:

(27a) K1,1=(eI+e1,1u)*e1,1De1,1ue1,1u*e1,1P*¯

(27b) K1,2=(e1,1BP+e1,2u)*e1,2De1,2ue1,2u*e1,2P*¯

(27c) Ki,1=(ei,i−1BP+ei,1u)*ei,1Dei,1uei,1u*ei,1P*¯ ;i∈{2,…,n}

(27d) Ki,2=(ei,iBP+ei,2u)*ei,2Dei,2uei,2u*ei,2P*¯ ;i∈{2,…,n}

(27e) Kn+1,1=(en+1,nBP+en+1,1u)*en+1,1Den+1,1uen+1,1u*en+1,1P*¯

(27f) Kn+1,2=(en+1,1P+en+1,3u)*en+1,3Den+1,3uen+1,3u*en+1,3P*¯

(27g) Kn+1,λ=(en+1,λP+en+1,λ+1u)*en+1,λ+1Den+1,λ+1uen+1,λ+1u*en+1,λ+1P*¯; λ∈{3,…,m−1}

(27h) Kn+1,m=(en+1,mP+en+1,2u)*en+1,2Den+1,2uen+1,2u*en+1,2P*¯

It is observed that Ki,j⊆Lm(Gi,j), where i∈{1,…,n+1} and j∈J(i). 

For $B_{i}$, where i∈{1,…,n}, the combination of the first and second rule is expressed in the form of two specifications. The first specification is as follows: once $R_{i}$ drops a product to $B_{i}$, then the product can be picked only by $R_{i+1}$. Τhe second specification is as follows: once $R_{i+1}$ drops a product to $B_{i}$, then the product can be picked only by $R_{i}$. For the two specifications, the corresponding regular languages are:

(28) Ki=ci¯; i∈{1,…,n}, KiB=ciB¯; i∈{2,…,n+1}

where the regular expressions ci and ciB are

ci=(ei+1,iBP+ei+1,iBD)*(ei,1P(ei+1,iBP+ei+1,iBD+ei+1,2P)*(ei,iBD)(ei+1,iBD+ei+1,2P)*(ei+1,iBP)+          +ei+1,2P(ei+1,iBP+ei+1,2P)*(ei+1,iBD)(ei+1,iBP+ei+1,iBD+ei+1,2P)*(ei,iBP)),ciB=(ei−1,i−1BP+ei−1,i−1BD)*ei,2P(ei−1,1P+ei−1,i−1BP+ei−1,i−1BD)*(ei,i−1BD)(ei−1,1P+ei−1,i−1BD)*(ei−1,i−1BP)+              +ei−1,1P(ei−1,1P+ei−1,i−1BP)*(ei−1,i−1BD)(ei−1,1P+ei−1,i−1BP+ei−1,i−1BD)*(ei,i−1BP)*.

Regarding the third rule, namely the coordination of the production sequence through the control of the robotic manipulators, the desired behavior of each robotic manipulator is expressed by the following regular languages:

(29a) K1R=eIe1,1D+e1,1Pe1,1BD+e1,1BPe1,2D+e1,2PeO*

(29b) KiR=ei,i−1BPei,1D+ei,1Pei,iBD+ei,iBPei,2D+ei,2Pei,i−1BD*;  i∈{2,…,n},

(29c) Kn+1R=en+1,nBPen+1,1D+en+1,1Pen+1,3D++λ=3m−1(en+1,λPen+1,λ+1D)+en+1,mPen+1,2D+en+1,2Pen+1,nBD*.

In the formulation of the third rule as the above set of regular languages, appropriate characteristics of the fourth and fifth rules have been used. It is observed that KiR⊆Lm(GiR) and the alphabet of KiR is the alphabet EiR, where i∈{1,…,n+1}.

**Proposition** **1.** *The regular language* KiR*, where* i∈{1,…,n+1}*, is* Lm(GiR)*-closed.*

**Proof of Proposition** **1.** According to [15,16], the language KiR is Lm(GiR)-closed if KiR=KiR¯∩Lm(GiR). Clearly, every word of KiR¯, not belonging to KiR, ends with a pick event. Furthermore, in every word of Lm(GiR), after a pick event, a drop event follows. This drop event activates the transition of GiR to its initial state, being the only marked state of GiR. Thus, KiR¯∩Lm(GiR)⊆KiR. Since, KiR⊆KiR¯ and KiR⊆Lm(GiR), the proof has been completed. □

The fourth rule is expressed by the following regular language:

(30) Ki,1D=(ei+1,1P*ei,1Pei+1,1P)*¯; i∈{1,…,n}

The fifth rule is expressed by the following regular language:

(31) Ki,2D=(ei−1,2P*ei,2Pei−1,2P)*¯; i∈{2,…,n+1}

Overall and mainly due to the second rule, the presented design specifications are less permissive than the design specifications proposed in [7] for the wafer manufacturing system with n=4 and m=3. It is noted that in such production lines, blocking can take place in cases where there are more products to be transported to the next stations and buffers than their number. In [7], appropriate coordination supervisors are proposed to avoid blocking. Each coordinator is essentially a product counter controlling only one robotic manipulator. In the present work, blocking is avoided using an alternative approach where, through the second rule, the robotic manipulator is permitted to receive products from a station only if the destination buffer and the respective production station following the buffer are empty. 

## 4. Distributed Supervisory Control Architecture 

The control of the wafer manufacturing process will be implemented by local control units, e.g., programmable logic controllers (PLCs), remote terminal units (RTUs), and microcontrollers. Each local control unit is denoted as $U_{i}$, where i∈{1,…,n+1}. Each $U_{i}$ is dedicated to controlling the allowability of the influence of a particular set of controllable events. This event set is disjoint to the respective set of any other local control unit. This way, inspired by the respective decentralized architecture proposed in [7] for n=4 and m=3, a distributed control configuration is proposed. Details regarding distributed supervisory control can be found in [15]. In Section 5, the design specifications, defined by the regular languages presented in Section 3.2, will be realized through appropriate supervisor automata implemented to the local control units $U_{i}$, where i∈{1,…,n+1}. 

Particularly, the supervisor automata implemented in $U_{i}$, where i∈{1,…,n}, are the supervisors Si,1, Si,2, Si, SiB, SiR,Si,1D, and Si,2D. The supervisors realize the languages Ki,1, Ki,2, Ki, KiB, KiR, Ki,1D, and Ki,2D, respectively. Note that the languages K1B and K1,2D do not correspond to any rule of desired behavior and are introduced exclusively for uniformity reasons to be K1B=E1R* and K1,2D=E1R*. 

The input signals of $U_{i}$, where i∈{1,…,n}, are:

(i) The events of $R_{i}$;
(ii) the single uncontrollable event of $C_{i,j}$, namely the event ei,ju, where j∈{1,2};
(iii) The events ei+1,iBP, ei+1,iBD, ei+1,1P, and ei+1,2P, of $R_{i+1}$;
(iv) the events ei−1,i−1BP, ei−1,i−1BD, ei−1,1P, and ei−1,2P of $R_{i-1}$, where i∈{2,…,n}.

Particularly, the supervisor automata implemented in $U_{n+1}$ are the supervisors Sn+1,1,…, Sn+1,m, Sn+1, Sn+1B, Sn+1R, Sn+1,1D, and Sn+1,2D. The supervisors realize the languages Kn+1,1,…,Kn+1,m, Kn+1, Kn+1B, Kn+1R, Kn+1,1D, and Kn+1,2D, respectively. Note that the languages Kn+1 and Kn+1,1D do not correspond to any rule of desired behavior and are introduced exclusively for uniformity reasons to be Kn+1=En+1R* and Kn+1,1D=En+1R*.

The input signals of $U_{n+1}$ are:

(i) The events of $R_{n+1}$;
(ii) The uncontrollable events of $C_{n+1,j}$, namely the events en+1,ju, where j∈{1,…,m};
(iii) The events en,nBP, en,nBD, en,1P, and en,2P of $R_{n}$.

Clearly, the events of the categories (iii) and (iv) of $U_{i}$, where i∈{1,…,n}, and the events of the category (iii) of $U_{n+1}$ cannot be restricted by the control unit and serve only as monitoring events. The events ei,ju, where i∈{1,…,n+1} and j∈J(i), being uncontrollable events of the production stations, cannot be restricted by the control unit $U_{i}$. Furthermore, the events ei+1,iBP, ei+1,iBD, ei+1,1P, ei+1,2P, and the events ei−1,i−1BP, ei−1,i−1BD, ei−1,1P, and ei−1,2P, where i∈{2,…,n+1}, are used by $U_{i}$ exclusively for monitoring purposes and are not allowed to be restricted by the control unit $U_{i}$. Based on this interconnection, the alphabet of $U_{i}$ will be augmented as compared to the alphabet of the automaton GiR. The augmented alphabet is the following:

(32) E˜iR=EiR∪{ei+1,iBP,ei+1,iBD,ei+1,1P,ei+1,2P,ei−1,i−1BP,ei−1,i−1BD,ei−1,1P,ei−1,2P,ei,1u,ei,2u}

where i∈{1,…,n}, and

(33) E˜n+1R=En+1R∪{en,nBP,en,nBD,en,1P,en,2P}∪j=1m{en+1,ju}

It is important to mention that the set of the events that can be restricted by $U_{i}$ (controllable event set), where i∈{1,…,n+1}, is E˜iR,u=EiR. Regarding these event sets, it holds that

(34a) E˜1R,u∩E˜kR,u=∅, ∀k∈{3,…,n+1}

(34b) E˜iR,u∩E˜kR,u=∅, ∀i,k∈{2,…,n} :k>i+1∧k<i−1

(34c) E˜n+1R,u∩E˜kR,u=∅, ∀k∈{1,…,n−1}

Also, regarding the adjacent control units, it holds that

(35a) E˜iR∩E˜i+1R={ei+1,iBP,ei+1,iBD,ei+1,1P,ei+1,2P}; i∈{1,…,n}

(35b) E˜iR∩E˜i−1R={ei−1,i−1BP,ei−1,i−1BD,ei−1,1P,ei−1,2P}; i∈{2,…,n+1}

Before presenting the design requirements of the present supervisory control scheme, the alphabets of the desired languages will be presented. The alphabets of K1,1 and K1,2 are E˜1,1=E1,1∪{eI} and E˜1,2=E1,2∪{eO}, respectively. The alphabets of Ki,1 and Ki,2 are E˜i,1=Ei,1∪{ei,i−1BP} and E˜i,2=Ei,2∪{ei,iBP}, where i∈{2,…,n}, respectively. The alphabet of Kn+1,1 is E˜n+1,1=En+1,1∪{en+1,nBP}, the alphabet of Kn+1,2 is E˜n+1,2=En+1,3∪{en+1,1P}, the alphabet of Kn+1,λ is E˜n+1,λ=En+1,λ+1∪{en+1,λP}, where λ∈{3,…,m−1}, and the alphabet of Kn+1,m is E˜n+1,m=En+1,2∪{en+1,mP}. The alphabet of Ki is E˜i={ei,iBP,ei,iBD,ei,1P,ei+1,2P,ei+1,iBP,ei+1,iBD}, where i∈{1,…,n}. The alphabet of Kn+1 is E˜n+1=En+1R. The alphabet of KiB is E˜iB={ei,i−1BP,ei,i−1BP,ei,2P,ei−1,1P,ei−1,i−1BP,ei−1,i−1BD}, where i∈{2,…,n+1}. The alphabet of K1B is E˜1B=E1R. The alphabet of KiR is EiR, where i∈{1,…,n+1}. The alphabet of Ki,1D is E˜i,1D={ei,1P,ei+1,1P}, where i∈{1,…,n}. The alphabet of Kn+1,1D is E˜n+1,1D=En+1R. Finally, the alphabet of Ki,2D is E˜i,2D={ei−1,2P,ei,2P}, where i∈{2,…,n+1}. The alphabet of K1,2D is E˜1,2D=E1R.

The design requirements of the supervisors of $U_{i}$, where i∈{1,…,n}, are expressed in terms of a respective controlled automaton, denoted by Gic, being of the following structure:

(36) Gic=GI||GO||GiR||Gi,1||Gi,2||Gi||Gi−1||SiR||Si,1||Si,2||Si||SiB||Si,1D||Si,2D==GiR||Gi,1||Gi,2||Gi||Gi−1||SiR||Si,1||Si,2||Si||SiB||Si,1D||Si,2D

where || denotes the synchronous product [15] (or the parallel connection [16]) of two automata, and where G0=({q1},∅,∅,∅,q1,{q1}). Clearly, for every automaton G it holds that G0||G=G. It is important to mention that the final equality in (36) results from the structure of the automata of the loading docks, having only one state and being marked, and a self-transition activated by all events of the automaton’s alphabet. The closed and the marked behavior of the synchronous product in (36), where i∈{1,…,n}, are in the following form:

(37a) L(Gic)=PiR−1 L(GiR)∩L(SiR)∩Pi−1 L(Gi)∩PiB−1 L(Gi−1)∩P˜i−1L(Si)∩P˜iB−1L(SiB)∩                ∩j=12Pi,j−1 L(Gi,j)∩P˜i,j−1L(Si,j)∩Pi,jD−1L(Si,jD),

(37b) Lm(Gic)=PiR−1 Lm(GiR)∩Lm(SiR)∩Pi−1 Lm(Gi)∩PiB−1 Lm(Gi−1)∩P˜i−1Lm(Si)∩P˜iB−1Lm(SiB)∩                   ∩j=12Pi,j−1 Lm(Gi,j)∩P˜i,j−1Lm(Si,j)∩Pi,jD−1Lm(Si,jD),

where PiR is the projection of E˜iR∗ to EiR∗; Pi,j and P˜i,j are the projections of E˜iR∗ to Ei,j∗ and E˜i,j∗, respectively; Pi and P˜i are the projections of E˜iR∗ to Ei∗ and E˜i∗, respectively; PiB and P˜iB are the projections of E˜iR∗ to E˜i−1∗ and E˜iB∗, respectively; Pi,1D is the projection of E˜iR∗ to E˜i,1D∗; and Pi,2D is the projection of E˜iR∗ to E˜i,2D∗. 

For i=n+1, the controlled automaton is required to be of the following structure:

(38) Gn+1c=Gn+1R||||j=1m(Gn+1,j||Sn+1,j)||Gn||Sn+1R||Sn+1B||Sn+1,2D

Clearly, the closed and the marked behavior of the synchronous product in (38) are in the following form:

(39a) L(Gn+1c)=Pn+1R−1 L(Gn+1R)∩L(Sn+1R)∩Pn+1B−1 L(Gn)∩P˜n+1B−1L(Sn+1B)∩                    ∩j=1mPn+1,j−1L(Gn+1,j)∩P˜n+1,j−1L(Sn+1,j)∩Pn+1,2D−1L(Sn+1,2D),

(39b) Lm(Gn+1c)=Pn+1R−1 Lm(Gn+1R)∩Lm(Sn+1R)∩Pn+1B−1 Lm(Gn)∩P˜n+1B−1Lm(Sn+1B)∩                     ∩j=1mPn+1,j−1Lm(Gn+1,j)∩P˜n+1,j−1Lm(Sn+1,j)∩Pn+1,2D−1Lm(Sn+1,2D),

where Pn+1R is the projection of E˜n+1R∗ to En+1R∗; Pn+1,j and P˜n+1,j are the projections of E˜n+1R∗ to En+1,j∗ and E˜n+1,j∗ (∀j∈J(i)), respectively; Pn+1B and P˜n+1B are the projections of E˜n+1R∗ to E˜n+1∗ and E˜n+1B∗, respectively; and Pn+1,2D is the projection of E˜n+1R∗ to E˜n+1,2D∗. 

In accordance with [7,16], the present supervisors are designed in a way that all supervisors’ states are marked. Thus, the closed and the marked behaviors of the supervisors for i∈{1,…,n+1} and j∈J(i) are required to be

(40a) L(Si,j)=Lm(Si,j)=Ki,j¯=Ki,j

(40b) L(Si)=Lm(Si)=Ki¯=Ki

(40c) L(SiB)=Lm(SiB)=KiB¯=KiB

(40d) L(Si,1D)=Lm(Si,1D)=Ki,1D¯=Ki,1D

(40e) L(Si,2D)=Lm(Si,2D)=Ki,2D¯=Ki,2D

(40f) L(SiR)=Lm(SiR)=KiR¯

According to Equation (40a–f), the closed and marked behaviors of the respective supervisors are designed to be equal to the prefix closure of the desired languages. Furthermore, regarding Equation (40a–e), it is observed that since the languages Ki,j, Ki, KiB, Ki,1D, and Ki,2D are prefix-closed, then the equality between the closed and marked behaviors of the respective supervisors is derived. Here, it is important to recall that, according to Proposition 1, the language KiR is Lm(GiR)-closed. For the definition and properties of prefixed-closed languages, see [15,16]. 

In addition to the above design requirements, the marked behavior of the controlled automaton Gic, for i∈{1,…,n}, is required to be

(41) Lm(Gic)=∩j=12Pi,j−1(Ki,j)∩Pi,jD−1(Ki,jD)∩PiR−1 (KiR)∩Pi−1(Ki)∩PiB−1(KiB)

For i=n+1, the marked behavior of the controlled automaton Gn+1c is required to be

(42) Lm(Gn+1c)=∩j=1mPn+1,j−1(Kn+1,j)∩Pn+1R−1 (Kn+1R)∩Pn+1B−1(Kn+1B)∩Pn+1,2D−1(Kn+1,2D)

In addition, for all i∈{1,…,n+1}, the marked behaviors of the controlled automata are required to satisfy the nonblocking requirement, i.e.,

(43) L(Gic)=Lm(Gic)¯

Finally, it is required for the supervisors embedded in $U_{i}$, where i∈{1,…,n+1} and j∈J(i), to be physical realizable (PR) (see [17]) with respect to the automata GiR, Gi,j, Gi, and Gi−1 through the synchronous products in (36) and (38).

The satisfaction of the design requirements (36), (38) and (40)–(43) will be accomplished through the distributed control architecture proposed here, providing separate development and implementation of the control algorithm of each control unit $U_{i}$. Furthermore, the control algorithm of each $U_{i}$ is analyzed for the conjunction of several supervisors providing modularity at the local control layer. The distributed- control architecture, designed for the present wafer system and being in accordance with the definition of distributed architectures presented in [7], is analyzed for a set of local controllers, i.e., one controller per robotic manipulator and the respective served production stations. Each local controller is fed by the events of the respective controlling subsystem and a set of distinct events coming from the adjacent subsystems through a “transfer line” (shared resources). It is mentioned that in [3,4], a distributed control architecture is proposed for the case where n=3 and m=3. To the best to our knowledge the presents control architecture is the first distributed control architecture for the general parametric model of wafer manufacturing systems.

**Figure 10:**
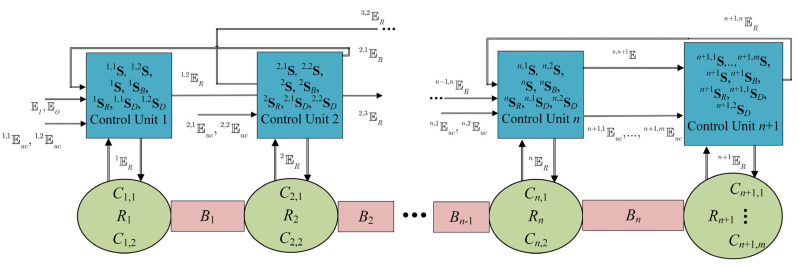
Distributed supervisory control architecture.

In Figure 10, a simplified drawing of the configuration of the distributed control architecture is presented. Ιn each control unit, the respective supervisors to be implemented and the respective participating alphabets are depicted. Clearly, each of the supervisors Sn+1, S1B, Sn+1,1D, and S1,2D, introduced for uniformity purposes, can be realized by an automaton with one state being marked and self-transitions for all the events of the corresponding alphabets. Nevertheless, the implementation of these supervisors is not necessary as they do not impose any additional desired performance. However, in the analysis in Section 6, they will be used for uniformity reasons.

As already mentioned, in the special case where n=4 and m=3, the resulting model of the system is similar to the one in [7]. In Section 3, differences and similarities of the proposed desired behavior, in comparison to the one in [7], have been presented. The main difference is imposed by the combination of the second, fourth, and fifth specifications, regarding blocking avoidance. In the present paper, a more restrictive rule is proposed. Particularly, if a buffer or a manufacturing station (in any flow direction) is full, then the respective robotic manipulator is not allowed to pick a product from the respective station. 

The present method can be effective in cases of station faults, i.e., if a fault takes place in a station, then the robotic manipulator is not allowed to pick or place products in this station. This way, the respective buffers are kept available to receive products from the reverse product flow.

The realization of the supervisors, depicted in Figure 10 and satisfying the design requirements (40a–f), will be determined in Section 5. In Section 6, the design requirements (40)–(43) and the physical realizability of the distributed supervisor design scheme will be proven to be satisfied. 

## 5. Supervisor Realization

### 5.1. Supervisors of the Production Stations 

The supervisors realizing the languages Ki,1, where i∈{1,…,n}, are in a 6-tuple form (see [17,18,19,20,21]), as follows:

(44) Si,1=(ℚi,1S,E˜i,1,fi,1S,ℍi,1S,xi,1S,0,ℚi,1S,m)

The set of the states of the supervisor automaton is ℚi,1S={qi,1S,1,qi,1S,2,qi,1S,3}. The initial state is xi,1S,0=qi,1S,1. The set of the marked states is ℚi,1S,m=ℚi,1S. The values of the transition functions of the supervisor automaton are

fi,1S(qi,1S,1,ei,i−1BP)=qi,1S,1, fi,1S(qi,1S,1,ei,1u)=qi,1S,1, fi,1S(qi,1S,1,ei,1D)=qi,1S,2, fi,1S(qi,1S,2,ei,1u)=qi,1S,3, fi,1S(qi,1S,3,ei,1P)=qi,1S,1

The active events sets are

ℍi,1S(qi,1S,1)={ei,i−1P,ei,1D,ei,1u}, ℍi,1S(qi,1S,2)={ei,1u}, ℍi,1S(qi,1S,3)={ei,1P,ei,1u}

**Figure 11:**
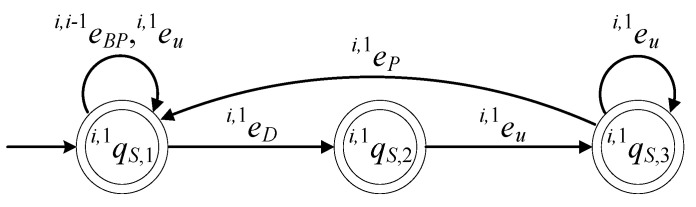
State diagram of supervisor Si,1.

The closed behavior and the marked behavior of the automaton satisfy the requirements (40a). In Figure 11, the state diagram of the supervisor is presented.

The supervisor S1,1 realizing the language K1,1, the supervisor Si,2 realizing the language Ki,2, where i∈{1,…,n}, and the supervisor Sn+1,j realizing Kn+1,j, where j∈{1,…,m}, have the same state diagram configuration as that in Figure 11. 

The supervisor automaton realizing Ki,1D, where i∈{1,…,n}, is the 6-tuple

(45) Si,1D=(ℚi,1D,S,Ei,1D,fi,1D,S,ℍi,1D,S,xi,1D,S,0,ℚi,1D,S,m)

The set of the states of the supervisor automaton is ℚi,1D,S={qi,1D,S,1,qi,1D,S,2}. The initial state is xi,1D,S,0=qi,1D,S,1. The set of the marked states is ℚi,1D,S,m=ℚi,1D,S. The values of the transition function of the supervisor automaton are

fi,1D,S(qi,1D,S,1,ei+1,1P)=qi,1D,S,1, fi,1D,S(qi,1D,S,1,ei,1P)=qi,1D,S,2, fi,1D,S(qi,1D,S,2,ei+1,1P)=qi,1D,S,1

The active events sets are

ℍi,1D,S(qi,1D,S,1)={ei,1P,ei+1,1P}, ℍi,1D,S(qi,1D,S,2)={ei+1,1P}

**Figure 12:**
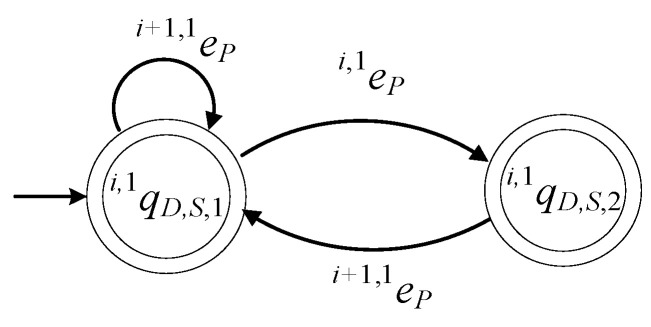
State diagram of supervisor Si,1D.

The closed behavior and the marked behavior of the automaton satisfy the requirements (40d). In Figure 12, the state diagram of the supervisor is presented.

The supervisor Si,2D of the language Ki,2D, where i∈{2,…,n+1}, has the same state diagram configuration as that in Figure 12. It is mentioned that a realization of the supervisors Sn+1,1D and S1,2D has already been proposed in Section 4. 

### 5.2. Supervisors of the Buffers

The supervisors for the buffers, namely the supervisors realizing the language Ki, where i∈{1,…,n}, are in the form

(46) Si=(ℚiS,E˜i,fiS,ℍiS,xiS,0,ℚiS,m)

The set of the states is ℚiS={qiS,1,qiS,2,qiS,3,qiS,4,qiS,5}. The initial state is xiS,0=qiS,1. The set of the marked states is ℚiS,m=ℚiS. The values of the transition functions are

fiS(qiS,1,ei+1,iBP)=qiS,1, fiS(qiS,1,ei+1,iBD)=qiS,1, fiS(qiS,1,ei,1P)=qiS,2,fiS(qiS,1,ei+1,2P)=qiS,4, fiS(qiS,2,ei+1,iBP)=qiS,2, fiS(qiS,2,ei+1,iBD)=qiS,2, fiS(qiS,2,ei+1,2P)=qiS,2, fiS(qiS,2,ei,iBD)=qiS,3, fiS(qiS,3,ei+1,iBP)=qiS,3, fiS(qiS,3,ei+1,2P)=qiS,3, fiS(qiS,3,ei+1,iBD)=qiS,1, fiS(qiS,4,ei+1,iBP)=qiS,4, fiS(qiS,4,ei+1,2P)=qiS,4, fiS(qiS,4,ei+1,iBD)=qiS,5, fiS(qiS,5,ei+1,iBP)=qiS,5, fiS(qiS,5,ei+1,iBD)=qiS,5, fiS(qiS,5,ei+1,2P)=qiS,5, fiS(qiS,5,ei,iBP)=qiS,1

The active events sets are

ℍiS(qiS,1)={ei,1P,ei+1,2P,ei+1,iBP,ei+1,iBD}, ℍiS(qiS,2)={ei,iBD,ei+1,2P,ei+1,iBP,ei+1,iBD}ℍiS(qiS,3)={ei+1,2P,ei+1,iBP,ei+1,iBD}, ℍiS(qiS,4)={ei+1,2P,ei+1,iBP,ei+1,iBD}, ℍiS(qiS,5)={ei,iBP,ei+1,2P,ei+1,iBP,ei+1,iBD}

**Figure 13:**
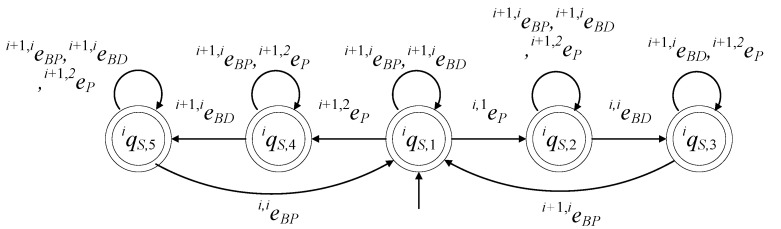
State diagram of supervisor Si.

The closed behavior and the marked behavior of the automaton satisfy the requirements (40b). In Figure 13, the state diagram of the supervisor is presented.

**Figure 14:**
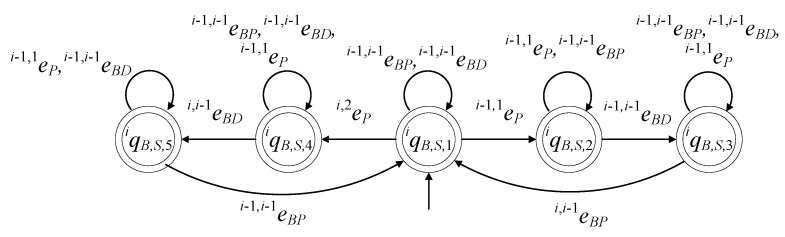
State diagram of supervisor SiB.

The state diagram of the supervisor SiB, where i∈{2,…,n+1}, has the same configuration as the state diagram of Si, where i∈{1,…,n}. The closed behavior and the marked behavior of the automaton satisfy the requirements (6c). In Figure 14, the state diagram of the supervisor SiB, where i∈{2,…,n+1}, is presented.

It is mentioned that the supervisors Sn+1 and S1B, mentioned in Section 4 to be supervisors realizing the languages Kn+1 and K1B, respectively, are single-state automata, with self-transitions activated by all events of the alphabet En+1R and the alphabet E1R, respectively. It is noted that the single states of the supervisor automata are marked. 

### 5.3. Supervisors of the Robotic Manipulators

The supervisor of the robotic manipulator $R_{i}$, namely the supervisor realizing the language KiR, where i∈{2,…,n}, is in the form

(47) SiR=(ℚiR,S,EiR,fiR,S,ℍiR,S,xiR,S,0,ℚiR,S,m)

The set of the states is ℚiR,S={qiR,S,1,qiR,S,2,qiR,S,3,qiR,S,4,qiR,S,5}. The initial state is xiR,S,0=qiR,S,1. The set of the marked states is ℚiR,S,m=ℚiR,S. The values of the transition function of the supervisor automaton are

fiR,S(qiR,S,1,ei,i−1BP)=qiR,S,2, fiR,S(qiR,S,1,ei,1P)=qiR,S,3fiR,S(qiR,S,1,ei,iBP)=qiR,S,4, fiR,S(qiR,S,1,ei,2P)=qiR,S,5, fiR,S(qiR,S,2,ei,1D)=qiR,S,1, fiR,S(qiR,S,3,ei,iBD)=qiR,S,1, fiR,S(qiR,S,4,ei,2D)=qiR,S,1, fiR,S(qiR,S,5,ei,i−1BD)=qiR,S,1

The active events sets are

ℍiR,S(qiR,S,1)={ei,i−1BP,ei,1P,ei,iBP,ei,2P},ℍiR,S(qiR,S,2)={ei ,1D}, ℍiR,S(qiR,S,3)={ei,iBD}, ℍiR,S(qiR,S,4)={ei,2D}, ℍiR,S(qiR,S,5)={ei,i−1BD}

**Figure 15:**
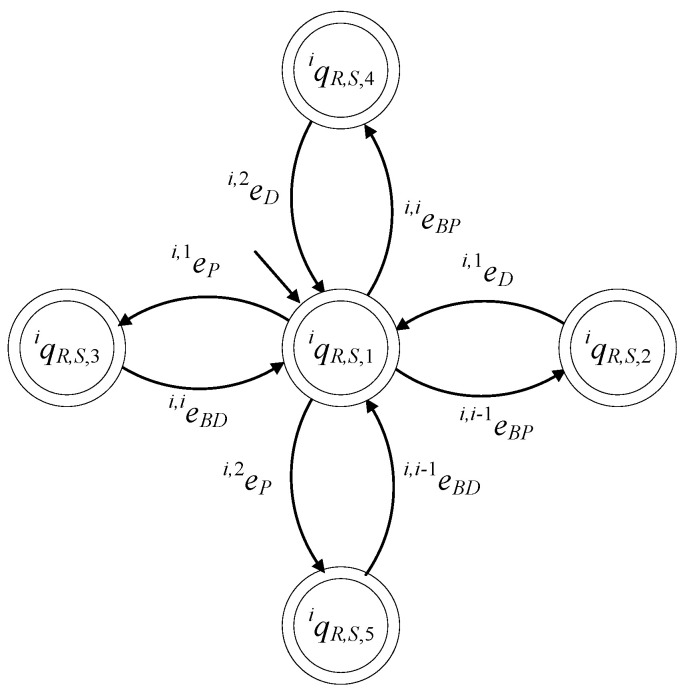
State diagram of robotic manipulator SiR.

The closed behavior and the marked behavior of the automaton satisfy the requirements (40f). In Figure 15, the state diagram of the supervisor is presented.

**Figure 16:**
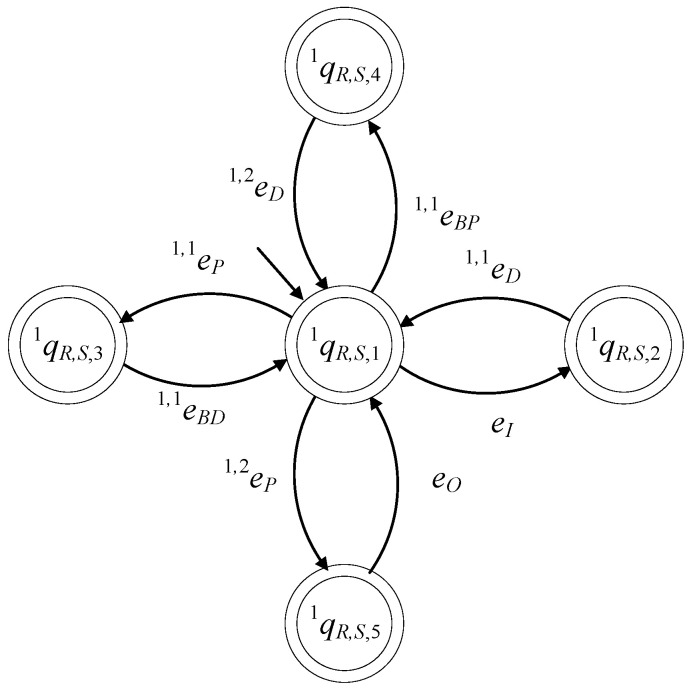
State diagram of robotic manipulator S1R.

The supervisor S1R of the language K1R has the same state diagram configuration as that in Figure 15. In Figure 16, the state diagram of the supervisor S1R is presented.

The supervisor of the robotic manipulator $R_{n+1}$, namely the supervisor realizing the language Kn+1R,D, is in the form

(48) Sn+1R=(ℚn+1R,S,En+1R,fn+1R,S,ℍn+1R,S,xn+1R,S,0,ℚn+1R,S,m)

The set of the states is ℚn+1R,S={qn+1R,S,1,qn+1R,S,2,…,qn+1R,S,m+2}. The initial state is xn+1R,S,0=qn+1R,S,1. The set of the marked states is ℚn+1R,S,m=ℚn+1R,S. The values of the transition functions of the supervisor automaton are

fn+1R,S(qn+1R,S,1,en+1,nBP)=qn+1R,S,2, fn+1R,S(qn+1R,S,1,en+1,1P)=qn+1R,S,3,fn+1R,S(qn+1R,S,1,en+1,jP)=qn+1R,S,j+1, ∀j∈{3,4,…,m}fn+1R,S(qn+1R,S,1,en+1,2P)=qn+1R,S,m+2, fn+1R,S(qn+1R,S,2,en+1,1D)=qn+1R,S,1,fn+1R,S(qn+1R,S,j,en+1,jD)=qn+1R,S,1,∀j∈{3,4,…,m}, fn+1R,S(qn+1R,S,m+1,en+1,2D)=qn+1R,S,1, fn+1R,S(qn+1R,S,m+2,en+1,nBD)=qn+1R,S,1

The active events sets are

ℍn+1R,S(qn+1R,S,1)={en+1,nBP,en+1,1P,…,en+1,mP}ℍn+1R,S(qn+1R,S,2)={en+1,1D}ℍn+1R,S(qn+1R,S,j)={en+1,jD},∀j∈{3,4,…,m},ℍn+1R,S(qn+1R,S,m+1)={en+1,2D}ℍn+1R,S(qn+1R,S,m+2)={en+1,nBD}

**Figure 17:**
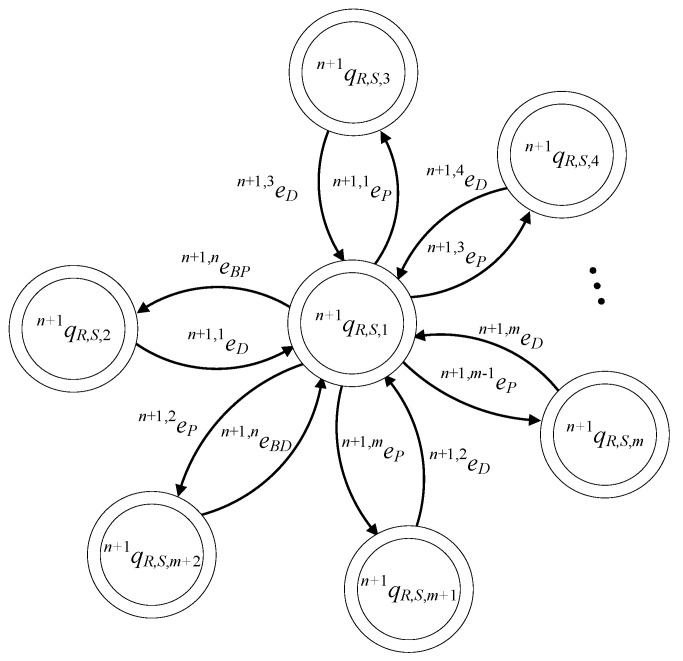
State diagram of robotic manipulator Sn+1R.

The closed behavior and the marked behavior of the automaton satisfy the requirements (40f). In Figure 17, the state diagram of the supervisor is presented.

## 6. Establishment of the Satisfactory Performance of the Controlled Automaton

In the present section, two lemmas, five theorems and two remarks regarding the implementability of the control architecture proposed in Section 4 will be established using the supervisor realizations derived in Section 5. In particular, the controllability of the desired languages will be proven through the proof of the physical realizability (see [17]) of the derived supervisors. Furthermore, the nonblocking property of the resulting controlled automaton will be proven. Finally, using these theorems and remarks, the satisfactory performance of the controlled automaton will be guaranteed. The satisfactory transition sequence of the controlled automaton will be determined in terms of the closed and the marked behavior of the controlled automaton. According to the distributed control architecture, proposed in Section 4, the supervisors embedded in $U_{i}$, where i∈{1,…,n+1}, are designed in order to satisfy the physical realizability of the supervisors with respect to the wafer manufacturing process, the nonblocking property of the resulting controlled automaton, and the satisfactory closed and marked behaviors of the controlled automaton. 

Regarding the physical realizability, the investigation is limited to the physical realizability of the supervisors of $U_{i}$ with respect to the robotic manipulator $R_{i}$, the production station $C_{i,j}$, where j∈J(i), and the buffer $B_{i}$, where i∈{1,…,n}, through the synchronous products in (36) and (38). The physical realizability of the supervisors of $U_{1}$ with respect to the loading docks is not investigated as all events in their alphabets are controllable. 

Regarding nonblocking, the investigation will start with the nonblocking property of the automata in the synchronous products (36) and (38). Then the nonblocking property of the overall system will be investigated. Regarding the loading docks, it is observed that they cannot cause blocking to the controlled automaton, as they have only one state, being marked, and a single event self-transition.

As already mentioned, in the present section, two lemmas and five theorems will be presented. In the two lemmas, the exclusion of non-marked states in appropriate synchronous products, due to non-accessibility, will be proven. Regarding non-accessible states, see [16]. In the first four theorems of the present section, the issues of physical realizability and nonblocking will be investigated with respect to the influence of the supervisors of $U_{i}$ to the devices controlled by the respective control unit. Finally, in the fifth theorem, the nonblocking property of the overall controlled wafer manufacturing system is investigated through the synchronous product of the participating controlled automata. 

In the first theorem, the physical realizability of the supervisors, embedded to $U_{i}$, where i={2,…,n}, with respect to the automata of the production stations, the robot, and the buffer affected by the events of the control unit $U_{i}$, through the synchronous product (36) will be proven. All automata affected by the control unit $U_{i}$ are composed as the synchronous product GiR||Gi||Gi−1||Gi,1||Gi,2. In the second theorem, the physical realizability of the supervisors, embedded to $U_{n+1}$, with respect to the automaton Gn+1R||Gn||j=1mGn+1,j, through the synchronous product (38) will be proven. Furthermore, the first of the two remarks of the section will cover the physical realizability of the supervisors, embedded to $U_{1}$.

**Theorem** **1.** *The synchronous product in (36), where* i={2,…,n}*, is physical realizable (PR), with respect to* GiR||Gi||Gi−1||Gi,1||Gi,2.

**Proof of Theorem** **1.** Regarding the supervisors embedded in the control unit $U_{i}$, the several events of the automaton GiR||Gi||Gi−1||Gi,1||Gi,2 are not allowed to be restricted by the supervisors. These events, being uncontrollable by $U_{i}$, are the uncontrollable events of the two production stations, i.e., the events ei,1u and ei,2u, and the monitoring events of the adjacent control units, i.e., the events ei+1,iBP, ei+1,2P, ei+1,iBD, ei−1,1P, ei−1,i−1BD, ei−1,i−1BP, ei+1,1P, and ei−1,2P. Here, it will be investigated if each supervisor of $U_{i}$ is able to restrict these 10 events. To this end, first it is observed that all events in the alphabet of SiR are controllable. Thus, SiR does not restrict any of the 10 events. Second, it is observed that the uncontrollable events in the alphabet of Si, being the events ei+1,iBP, ei+1,2P, and ei+1,iBD, satisfy the following properties:

ei+1,iBP∈∩λ=15ℍiS(qiS,λ), ei+1,2P∈∩λ=15ℍiS(qiS,λ), ei+1,iBD∈∩λ=13ℍiS(qiS,λ)

The above relations reveal that the three uncontrollable events of Si belong to the active even sets of all states of Si. Thus, Si does not restrict any of the 10 events. Third, it is observed that the uncontrollable events in the alphabet of SiB, being the events ei−1,1P, ei−1,i−1BD, and ei−1,i−1BP, satisfy the following properties

ei−1,1P∈∩λ=15ℍiB,S(qiB,S,λ),ei−1,i−1BD∈∩λ=15ℍiB,S(qiB,S,λ),ei−1,i−1BP∈∩λ=15ℍiB,S(qiB,S,λ)

The above relations reveal that the three uncontrollable events of SiB belong to the active even sets of all states of SiB. Thus, SiB does not restrict any of the 10 events.

Fourth, it is observed that there is only one uncontrollable event in the alphabet of Si,1D, being the event ei+1,1P, where ei+1,1P∈∩λ=12ℍi,1D,S(qi,1D,S,λ). The above relation reveals that the uncontrollable event of Si,1D belongs to the active even sets of all states of Si,1D. Thus, Si,1D does not restrict any of the 10 events. Fifth, it is observed that there is only one uncontrollable event in the alphabet of Si,2D, being the event ei−1,2P where ei−1,2P∈∩λ=12ℍi,2D,S(qi,2D,S,λ). The above relation reveals that the uncontrollable event of Si,2D belongs to the active even sets of all states of Si,2D. Thus, Si,2D does not restrict any of the 10 events. Sixth, it is observed that there is only one uncontrollable event in the alphabet of Si,j, where j∈J(i), being the event ei,ju where ei,ju∈∩λ=13ℍi,jS(qi,jS,λ). The above relation reveals that the uncontrollable event of Si,j belongs to the active even sets of all states of Si,j. Thus, Si,j does not restrict any of the 10 events. Hence, according to Corollary 1 in [17], all supervisors are PR with respect to GiR||Gi||Gi−1||Gi,1||Gi,2, through (36). □

**Remark** **1.** *The proof of the physical realizability for* $U_{1}$ *is similar to the one of* $U_{i}$*, for* i∈{2,…,n}*, if the events of the loading docks are considered, i.e., if the events* $e_{I}$ *and* $e_{O}$ *are used instead of the events* ei,i−1BP *and* ei,i−1BD *in the proof of Theorem 1.*

**Theorem** **2.** *The synchronous product in (38) is physically realizable (PR) with respect to* Gn+1R||Gn||j=1mGn+1,j.

**Proof of Theorem** **2.** The uncontrollable events of the automaton Gn+1R||Gn||j=1mGn+1,j are the m uncontrollable events of the last m production stations, i.e., the events ei,ju where j∈{1,…,m} and the monitoring events of the adjacent control units considered as uncontrollable events. The monitoring events are the events en,1P, en,2P, en,nBD, and en,nBP. Here, it will be investigated if each supervisor of $U_{n+1}$ is able to restrict these m+4 events. To this end, first, it is observed that all event of Sn+1R are controllable. Thus, Sn+1R does not restrict any of the m+4 events. Second, it is observed that the uncontrollable events in the alphabet of Sn+1B, being the events en,1P, en,nBD and en,nBP, satisfy the following properties:

en,1P∈∩λ=15ℍn+1B,S(qiB,S,λ), en,nBD∈∩λ=15ℍn+1B,S(qiB,S,λ), en,nBP∈∩λ=15ℍn+1B,S(qiB,S,λ)

The above relations reveal that the three uncontrollable events of Sn+1B belong to the active even sets of all states of Sn+1B. Thus, Sn+1B does not restrict any of the m+4 events. Third, it is observed that there is only one uncontrollable event in the alphabet of Sn+1,2D, being the event en,2P where en,2P∈∩λ=12ℍn+1,2D,S(qn+1,2D,S,λ). The above relation reveals that the uncontrollable event of Sn+1,2D belongs to the active even sets of all states of Sn+1,2D. Thus, Sn+1,2D does not restrict any of the m+4 events. Fourth, it is observed that there is only one uncontrollable event in the alphabet of Sn+1,j, being the event en+1,ju where en+1,ju∈∩λ=13ℍn+1,jS(qn+1,jS,λ). The above relation reveals that the uncontrollable event of Sn+1,j belongs to the active even sets of all states of Sn+1,j. Thus, Sn+1,j does not restrict any of the m+4 events. Hence, according to Corollary 1 in [17], all supervisors in (38) are PR with respect to Gn+1R||Gn||j=1mGn+1,j, through (38). Since the uncontrollable alphabets of all supervisors are disjoint sets, the conjunction of all supervisors is PR with respect to Gn+1R||Gn||j=1mGn+1,j, through (38). □

In the following two lemmas, a subautomaton of the synchronous product of the automaton of the production station and one of the proposed supervisors, as well as a subautomaton of the synchronous product of the automaton of the buffer and two of the proposed supervisors, are presented. All states of these two subautomata will be proven to be marked. Regarding subautomata, see [15,16].

**Lemma** **1.** *All states of the automaton* Gi,jc=Gi,j||Si,j*, for* i∈{1,…,n+1} *and* j∈J(i)*, are marked states*.

**Proof of Lemma** **1.** For the automaton Gi,jc it holds that

fi,jc(qi,jS,1,qi,j1),e=(qi,jS,1,qi,j1); e∈E˜i,j−Ei,j, fi,jc(qi,jS,1,qi,j1),ei,jD=(qi,jS,2,qi,j2),fi,jc(qi,jS,2,qi,j2),ei,ju=(qi,jS,3,qi,j3), fi,jc(qi,jS,3,qi,j3),ei,jP=(qi,jS,1,qi,j1)

where fi,jc(·,·) is the transition function and ℍi,jc(·) is the set of the active events of the automaton Gi,jc. From the above it is observed that any pair of states, where the first element is qi,j4 and the second is a state of Si,j, is not a state of Gi,jc, in the sense that this pair is non-accessible. The rest states of Gi,j, i.e., the states qi,j1, qi,j2, and qi,j3, are all marked. Furthermore, the states of Si,j are all marked. Thus, the proof has been completed. □

**Corollary** **1.** *The automaton* Gi,jc *is a nonblocking automaton*.

Before presenting the following lemma, it is important to mention that the alphabets of the buffers contain events of the adjacent control units, controlling the buffer. Thus, the controlled automaton of the buffer is a result of the joint action of the adjacent control units.

**Lemma** **2.** *All accessible states of the automaton* Gi*, under the influence of the supervisors* Si *and* Si+1B*, for* i∈{1,…,n}*, are marked states.*

**Proof of Lemma** **2.** First, the properties of the automaton Gi||Si will be examined. Regarding Gi, it is observed that the arrival to the non-marked state qi4, having no transitions, can be accomplished only by transitions from qi1, qi2, and qi3. Regarding qi1, the respective transitions in Gi||Si are

fiG||S(qi1,qiS,1),ei+1,iBP=(qi4,qiS,1),fiG||S(qi1,qiS,2),ei+1,iBP=(qi4,qiS,2),fiG||S(qi1,qiS,4),ei+1,iBP=(qi4,qiS,4)

where fiG||S(·,·) is the transition function of the automaton Gi||Si. Regarding qi2, the respective transition in Gi||Si is fiG||S(qi2,qiS,3),ei+1,iBD=(qi4,qiS,3). Regarding qi3, the respective transitions in Gi||Si are fiG||S(qi3,qiS,5),ei+1,iBD=(qi4,qiS,5) and fiG||S(qi3,qiS,5),ei+1,iBP=(qi4,qiS,5). At first, it is observed that Gi||Si is a blocking automaton, where all events provoking a transition to the non-marked state qi4 are events of $U_{i+1}$. Next, the properties of Gi||Si+1B will be examined. It is observed that the transitions to the non-marked state qi4, from the state qi1, are

fiG||B(qi1,qiB,S,1),ei,iBP=(qi4,qiS,1),fiG||B(qi1,qiB,S,2),ei,iBP=(qi4,qiB,S,2),fiG||B(qi1,qiB,S,4),ei,iBP=(qi4,qiB,S,4)

where fiG||B(·,·) is the transition function of the automaton Gi||Si+1B. The respective transitions from qi2 are

fiG||B(qi2,qiB,S,5),ei,iBP=(qi4,qiB,S,5), fiG||B(qi2,qiB,S,5),ei,iBD=(qi4,qiB,S,5)

The transition from qi3 is fiG||B(qi3,qiB,S,3),ei,iBP=(qi4,qiB,S,3). Thus, the automaton Gi||Si+1B is a blocking automaton, where all events provoking a transition to the non-marked state qi4 are events of $U_{i}$. From the above it is concluded that the joint action of the control units $U_{i}$ and $U_{i+1}$ to Gi restricts all transitions to qi4. Since, qi4 is the only non-marked state of Gi, it is concluded that Gi, under the influence of the supervisors Si and Si+1B, namely the resulting controlled automaton, has all its accessible states marked. Clearly, the controlled automaton is a nonblocking automaton. □

From Lemma 2 it is concluded that the non-marked state of the automaton of the buffer $B_{i}$ is non-accessible, under the influence of the control actions imposed by $U_{i}$ and $U_{i+1}$. Define the automaton G˜i, being a subautomaton of Gi and derived by removing the single non-marked state Gi and the corresponding transitions of Gi. Clearly, the set of the states of G˜i is the set ℚim. Using the present definition, the following synchronous products, being subautomata of the controlled automata in (36) and (38), are derived

(49a) G˜ic=GiR||Gi,1c||Gi,2c||G˜i||G˜i−1||SiR||Si||SiB||Si,1D||Si,2D, i∈{1,…,n}

(49b) G˜n+1c=Gn+1R||||j=1m(Gn+1,jc)||G˜n||Sn+1R||Sn+1B||Sn+1,2D

In the following two theorems, the property of nonblocking of G˜ic, where i={2,…,n}, and the nonblocking property of G˜n+1c will be proven. Following [15] (Section 4.9, p. 190), the proof will be accomplished by investigating if the controlled automaton always has an active transition from any non-marked state to a marked state. Finally, a remark, being the second remark of the present section, will cover the nonblocking property of G˜1c.

**Theorem** **3.** *The synchronous product in (49a), namely the controlled automaton* G˜ic*, where* i∈{2,…,n}*, is a nonblocking automaton.*

**Proof of Theorem** **3.** For the proof of Theorem 3, see Appendix A. □

**Remark** **2.** *The proof of the nonblocking property of* G˜1c *follows the one of Theorem 3, if the events of the loading docks are considered, i.e., if the events* $e_{I}$ *and* $e_{O}$ *are used instead of the events* ei,i−1BP *and* ei,i−1BD *in the proof of Theorem 3*.

**Theorem** **4.** *The synchronous product in (49b), namely the controlled automaton* G˜n+1c*, is a nonblocking automaton.*

**Proof of Theorem** **4.** For the proof of Theorem 4, see Appendix B. □

In the following theorem, the nonblocking property of the overall manufacturing system will be proven.

**Theorem** **5.** *The automaton* G1c||G2c||⋯||Gn+1c *is a nonblocking automaton*.

**Proof of Theorem** **5.** Consider the automata Gic and Gi+1c, where i∈{1,…,n}. Using Lemma 1, the synchronous product Gic||Gi+1c of the two automata is expressed as follows:

(50) Gic||Gi+1c=GiR||||j=1|J(i)|Gi,jc||Gi||Gi−1||SiR||Si||SiB||Si,1D||Si,2D||Gi+1R||                          ||j=1|J(i+1)|Gi+1,jc||Gi+1||Gi||Si+1R||Si+1||Si+1B||Si+1,1D||Si+1,2D.

where | · | denotes the cardinality of the argument set. Using the supervisors Si+1B and Si twice in the above formula, it is observed that the closed and the marked language of Gic||Gi+1c are equal to the closed and the marked language of the automaton:

(51) GiR||||j=1|J(i)|Gi,jc||Gi||Gi−1||SiR||Si||Si+1B||SiB||Si,1D||Si,2D||Gi+1R||                      ||j=1|J(i+1)|Gi+1,jc||Gi+1||Gi||Si+1R||Si+1||Si+1B||Si||Si+1,1D||Si+1,2D.

Using the remarks before relation (49a,b), it is observed that the closed and the marked behavior of the above automaton are equal to the closed and the marked behavior of the following automaton:

(52) GiR||||j=1|J(i)|Gi,jc||G˜i||Gi−1||SiR||Si||Si+1B||SiB||Si,1D||Si,2D||Gi+1R||                     ||j=1|J(i+1)|Gi+1,jc||Gi+1||G˜i||Si+1R||Si+1||Si+1B||Si||Si+1,1D||Si+1,2D.

Using (49a,b), it is observed that the closed and the marked behavior of G1c||G2c||⋯||Gn+1c are equal to the closed and the marked behavior of the subautomaton G˜1c||G˜2c||⋯||G˜n+1c. In Theorems 3 and 4, the nonblocking property of G˜ic, where i∈{1,…,n+1}, has been proved. Furthermore, in Theorems 1 and 2 it has been shown that all the events from the adjacent control units are considered as uncontrollable events, and they are not restricted by the respective supervisors. Thus, the supervisors of the control unit $U_{i}$ do not restrict the events of G˜ic, being common with G˜i−1c or G˜i+1c. Furthermore, G˜ic does not have any common event with the rest automata. Thus, the nonblocking property of G˜ic is not affected by the rest-controlled automata in the synchronous product G˜1c||G˜2c||⋯||G˜n+1c. Hence, G˜1c||G˜2c||⋯||G˜n+1c is a nonblocking automaton, and consequently G1c||G2c||⋯||Gn+1c is also a nonblocking automaton. □

In Theorems 1 and 2 and in Remark 1, the physical realizability of the supervisors implemented in $U_{i}$ with respect to the respective manufacturing devices related to $U_{i}$, namely the devices $B_{i}$, $R_{i}$ and $C_{i,j}$ where j∈J(i), through the respective synchronous products, has been proved. The events of the adjacent control units have been considered as uncontrollable events and they cannot be restricted by $U_{i}$. The latter implies that the events of $U_{i+1}$ used in $U_{i}$ have not been restricted and vice versa. Thus, the PR property of the synchronous product G1c||G2c||⋯||Gn+1c is derived.

From relations (40) and (49), as well as the five theorems and the remarks, presented above, it is concluded that the system performance of the controlled automaton Gic is satisfactory for every i∈{1,…,n+1}. Recall that the languages Ki,j are prefix-closed languages and P˜i,j−1Ki,j⊆Pi,j−1 L(Gi,j), ∀i∈{1,…,n+1}, and ∀j∈J(i), as well as that the languages KiR are Lm(GiR)-closed (see Proposition 1) and KiR⊆Lm(GiR), for every i∈{1,…,n+1}. Τhe satisfactory performance of the closed and the marked behaviors of the controlled automata, i.e., the satisfactory performance of the languages L(Gic) and Lm(Gic), for every i∈{1,…,n+1}, is validated by the following relations:

(53a) L(Gic)=L(G˜ic)=PiR−1L(GiR)∩PiR−1KiR¯∩Pi−1L(G˜i)∩PiB−1L(G˜i−1)∩    ∩j=1|J(i)|Pi,j−1L(Gi,j)∩P˜i,j−1Ki,j¯∩P˜i−1Ki¯∩P˜iB−1KiB¯∩∩j=12Pi,jD−1Ki,jD¯==PiR−1KiR¯∩P˜i−1Ki¯∩P˜iB−1KiB¯∩∩j=12Pi,jD−1Ki,jD¯∩∩j=1|J(i)|P˜i,j−1Ki,j¯,

(53b) Lm(Gic)=Lm(G˜ic)=PiR−1Lm(GiR)∩PiR−1KiR¯∩Pi−1 Lm(G˜i)∩PiB−1 Lm(G˜i−1)∩      ∩j=1|J(i)|P˜i,j−1Ki,j¯∩Pi,j−1 Lm(Gi,j)∩P˜i−1Ki¯∩P˜iB−1KiB¯∩∩j=12Pi,jD−1Ki,jD¯=      =PiR−1KiR∩P˜i−1Ki∩P˜iB−1KiB∩∩j=12Pi,jD−1Ki,jD∩∩j=1|J(i)|P˜i,j−1Ki,j,

where for the derivation of the above relations, the relations P˜i−1Ki⊆Pi−1 Lm(G˜i) and P˜iB−1Ki⊆PiB−1 Lm(G˜i−1) have been used. 

## 7. Simulation Results

To illustrate the performance of the closed and marked behaviors of the controlled automata of the proposed control scheme, simulation results for the controlled automata of the overall wafer manufacturing system will be presented. Consider the case of the wafer manufacturing process where n=4 and m=3. In addition, consider the case where the following concatenation of events took place:

eIe1,1De1,1Pe1,1ue1,1Pe1,1De1,1BDeIe2,1BPe1,1De2,1De1,1ue1,1Pe2,1ue2,1Pe1,1Pe2,1BDe1,1BD

The above event concatenation corresponds to a sequence of operation commands appropriately enriched with sensor signals. To interpret the above word, appropriate prefixes of the word will be analyzed. First, consider the prefix eIe1,1De1,1P. This prefix is analyzed as follows: $R_{1}$ is commanded to pick a product from the loading dock; $R_{1}$ is commanded to drop the product to $C_{1,1}$; then, $R_{1}$ is commanded to pick the product from $C_{1,1}$. The latter command would lead $C_{1,1}$ to malfunction, but the supervisor S1,1 has disactivated this command. Thus, the supervisor allows the station to manufacture the dropped product and the sensor signal, indicating that $C_{1,1}$ finished the manufacturing of the product, to take place. Thus, the prefix eIe1,1De1,1Pe1,1u has been interpreted. Continuing, the prefix eIe1,1De1,1Pe1,1ue1,1Pe1,1De1,1BD is analyzed as follows: After the sensor signal, indicating that $C_{1,1}$ finished the manufacturing of the product, $R_{1}$ is commanded to pick the product from $C_{1,1}$. After, $R_{1}$ is commanded to drop the product again to $C_{1,1}$. However, the latter command is disabled by the supervisors S1 and S1R. So, the next command, namely the command to drop the product to $B_{1}$, is executed. Continuing with the prefix eIe1,1De1,1Pe1,1ue1,1Pe1,1De1,1BDeIe2,1BPe1,1De2,1D, the remaining commands of the prefix are analyzed as follows: $R_{1}$ is commanded to pick another product from the loading dock; $R_{2}$ is commanded to pick a product from $B_{2}$; $R_{1}$ is commanded to drop a product to $C_{1,1}$; $R_{2}$ is commanded to drop a product to $C_{2,1}$. Since $C_{1,1}$ has already finished the manufacturing of the product, $R_{1}$ is commanded to pick the product from $C_{1,1}$, but the supervisor S1,1D has disactivated the command e1,1P till $C_{2,1}$ is empty. Thus, the prefix eIe1,1De1,1Pe1,1ue1,1Pe1,1De1,1BDeIe2,1BPe1,1De2,1De1,1u has been interpreted. After $C_{2,1}$ finished the manufacturing of the product, the remaining commands of the whole word are interpreted as follows: $R_{2}$ is commanded to pick a product from $C_{2,1}$, $R_{1}$ is commanded to pick a product from $C_{1,1}$, $R_{2}$ is commanded to drop a product to $B_{2}$, and $R_{1}$ is commanded to drop a product to $B_{1}$.

In Table 1, the influence of the previous events concatenation to the automata of the overall controlled wafer manufacturing system are presented. In the first column of Table 1, the events that take place are presented. In the next six columns of Table 1, the current states of the automata of the robotic systems $R_{1}$ and $R_{2}$, the production stations $C_{1,1}$ and $C_{2,1}$, and the buffers $B_{1}$ and $B_{2}$ are presented.

The colored rows in Table 1 indicate that an event that was restricted by the supervisors took place. According to the third row, the event e1,1P has been restricted to take place by the supervisor S1,1. According to the sixth row, the event e1,1D has been restricted to take place by the supervisors S1 and S1R. Finally, according to the 13th row, the event e1,1P has been restricted to take place by the supervisor S1,1D. The event e1,1P will not provoke a transition in the system until the event e2,1P takes place that empties the production machine $C_{2,1}$. As already mentioned in Section 3, the supervisor S1,1D is not included in the supervisory control scheme proposed in [7]. However, the decentralized architecture proposed in [7] guarantees that if there are free slots (buffers and/or production machines), after $C_{1,1}$ and $B_{1}$, then the product will eventually move on; a possible malfunction in the production machine $C_{2,1}$ may cause product blocking in the buffer $B_{1}$. 

## 8. Complexity of the Control Architecture 

The complexity of an automaton is described by the triad including the number of states, the number of events, and the number of transitions of the automaton; indicatively, see [21,22,23]. The complexity triads of Si,1 and Si,2, where i∈{1,…,n}, are equal to $\left(3,\;4,\;6\right)$. The complexity triad of Si, where i∈{1,…,n}, is equal to $\left(5,\;6,\;18\right)$. The complexity triad of SiB, where i∈{2,…,n+1}, is equal to $\left(5,\;6,\;18\right)$. The complexity triad of Si,1D, where i∈{1,…,n}, is equal to $\left(2,\;2,\;3\right)$. The complexity triad of Si,2D, where i∈{2,…,n+1}, is $\left(2,\;2,\;3\right)$. The complexity triad of SiR, where i∈{1,…,n}, is $\left(5,\;8,\;8\right)$. The complexity triad of the supervisor Sn+1R is $\left(m+2,2m+2,2m+2\right)$. Regarding the supervisors Sn+1,1D, S1, 2D, Sn+1, and S1B, it is mentioned that is not necessary to be realized. Hence, the total complexity triad of all supervisors in $U_{1}$ is computed to be $\left(18,\;24,\;39\right)$. The total complexity triad of all supervisors in $U_{i}$, where i∈{2,…,n}, is computed to be $\left(25,\;32\;,50\right)$. Similarly, the total complexity triad of all supervisors in $U_{n+1}$ is computed to be $\left(4m+9,6m+10,8m+23\right)$. Hence, the total complexity of the proposed distributed supervisor design scheme is computed to be $\left(25n+4m+2,\;32n+6m+2,\;50n+8m+12\right)$. It is mentioned that the total complexity of the proposed supervisory scheme is linear with respect to the number of robots and the number of stations. It is also mentioned that the complexity of the present wafer manufacturing process is equal to $\left(10n+4m+4,\;15n+5m+4,\;27n+9m+4\right)$. In comparison to the complexity of the wafer manufacturing process, it is observed that the complexity of the supervisor scheme proposed here is of analogous order of size. 

## 9. Implementation of the Distributed Supervisor Architecture

The problem of implementation of modular supervisory control schemes in PLCs is tackled in many articles; see [24,25]. In [21,22], the implementation of various modular supervisory control schemes to real-time industrial controllers, e.g., PLCs, has been presented using ladder diagrams following the international standard IEC 61131–3 (2013). The distributed supervisory architecture proposed here can be easily implemented following the directions of [22]. More specifically, each local control unit implements a specific number of supervisors with predefined complexity. The supervisors of each local control unit can be realized either by classic ladder diagrams or by function blocks using ladder diagrams or languages based on structured text. It is important to mention that, as shown in Section 5, many supervisors share a common structure. Hence, they can be easily implemented in the event-driven architecture of the IEC 61499 function blocks [26] with parametric input/output signals [27]. The above characteristic of the supervisory control architecture, proposed here, facilitates the implementation of the supervisors as they can easily be programmed in function blocks by developing one library for each supervisor. Clearly, for any possible extension of the manufacturing process, the respective control scheme can easily be implemented using these libraries.

**Figure 18:**
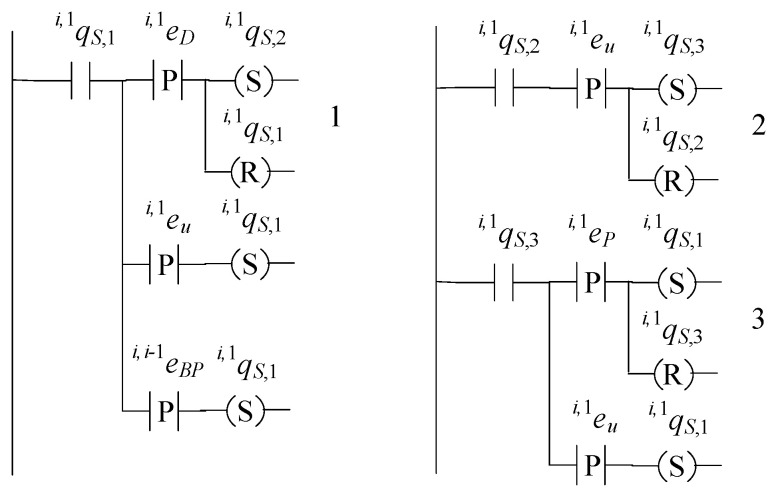
Ladder diagram of Si,1.

**Figure 19:**
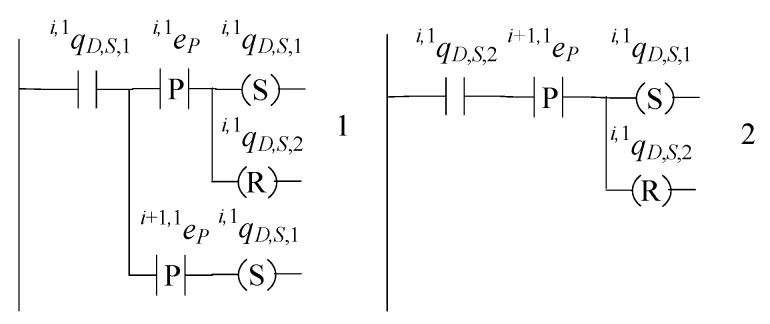
Ladder diagram of Si,1D.

**Figure 20:**
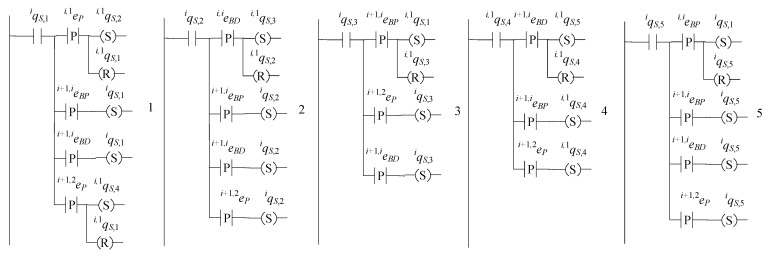
Ladder diagram of Si.

**Figure 21:**
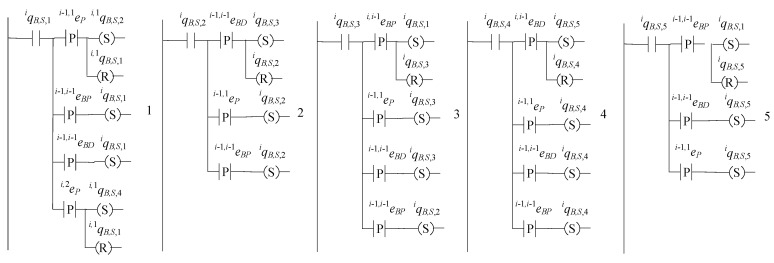
Ladder diagram of SiB.

**Figure 22:**
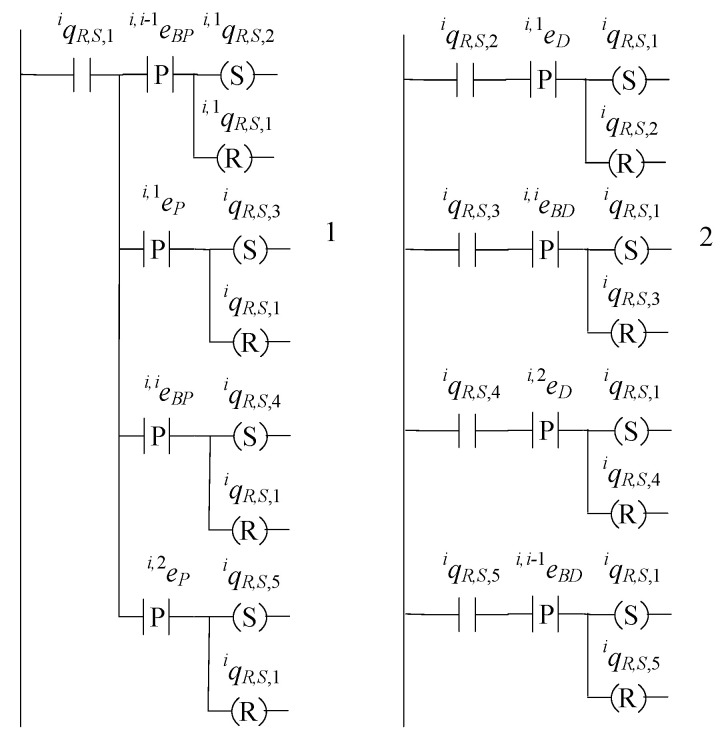
Ladder diagram of SiR.

In order to reveal the implementability of the present results, the supervisors embedded in $U_{i}$ are implemented, using the IEC 61131-3 (2013) [28] ladder diagram (LD) framework, shown in Figure 18, Figure 19, Figure 20, Figure 21 and Figure 22. The implementation is based on the respective state diagrams presented in Section 4. The ease of implementability of the present scheme is based on the explicit design of small-size supervisor automata. This is an advantage of the present results. In Figure 18, Figure 19, Figure 20, Figure 21 and Figure 22 the numbers at the side of each block, named rung, denote the transitions of each state.

As mentioned in Section 4, the state diagrams of S1,1, Si,2, and Sn+1,j, where i∈{1,…,n} and j∈{1,…,m}, have the same structure as the state diagram of Si,1. Hence, the LDs of S1,1, Si,2, and Sn+1,j, where i∈{1,…,n} and j∈{1,…,m}, have the same structure as the state diagram of Si,1 in Figure 18. Similarly, the LD of Si,2D, where i∈{2,…,n+1}, has the same structure as the LD of Si,1D in Figure 19. Finally, it is mentioned that the LDs of S1R and Sn+1R have the same structure as the LD of SiR in Figure 22.

According to [29], a distributed control architecture cannot be viewed only in a geographical point of view but also regarding the functionality of the system’s components. In order to impose the desired control actions [30], the control units of a distributed architecture communicate with each other and possibly with other external units. In Industry 4.0, the most common network for the interconnection of the distributed controllers is the ethernet network for Supervisory Control and Data Acquisition (SCADA) and Manufacturing Execution System (MES), which connects controllers, machines, and systems ([29,30,31]). Regarding PLC communication, a widely used network is the OPC UA as well as the Modbus protocol (see [32,33]). In the present control architecture, the resources of the control unit of each robotic manipulator are the signals of the previous and the next robotic manipulator as well as the two uncontrollable events (signals) of the production machines. All these signals, together with the commands of the respective robotic manipulator, are the signals used by the respective control unit and required from the design specifications of the supervisors implemented in the control unit. 

The control implementation directions, proposed above, facilitate the development of a control software tool that is dedicated to wafer manufacturing industries with no limitation to the number of vacuum chambers and consequently the number of robotic manipulators. The software offers itself for newly installed or extended wafer manufacturing plants. This can be accomplished due to the parametric and distributed structure of the control scheme. In simple words, the implementation is accomplished by embedding a copy of the local control software to any additional control unit. Therefore, the work of the programmer is limited mainly to the assignment of the parameters of the local control function blocks.

## 10. Conclusions

A modular parametric discrete event model with respect to the number of production stations, the number of buffers, and the number of robotic manipulators has been developed for a wafer manufacturing system. The parametric model has been built in a way that provides a clear direction towards the modelling of the continuously expandable wafer production industry. A set of desired specifications has been imposed in the form of five rules. A comparison between the proposed rules and those of the literature has been presented. Next, the desired specifications have been translated and decomposed to a set of local regular languages, one set per robotic manipulator. 

A distributed supervisory control architecture has been developed based on the local regular languages. In the proposed architecture, a set of local supervisors is designed for each robotic manipulator with appropriate signal resources shared between adjacent control units. 

Here, the PR properties of the developed supervisors have been proved regarding the total automaton of the system. The nonblocking property of the proposed architecture has been guaranteed. Finally, implementation issues have been tackled, and the complexity of the distributed architecture has been determined in a parametric formula.

The extension of the modelling and the distributed supervisory control architecture to wafer manufacturing systems with a parametric number of production stations served by the same robotic manipulator is currently under investigation. In addition, diagnosis and fault tolerance issues of the wafer manufacturing systems are under investigation. First, the fault tolerance characteristics of the proposed control scheme will be investigated in the cases of sensors and actuator faults as well as the resilience of the control scheme in the presence of cyberattacks. Second, extensions of the proposed control scheme towards improving tolerance will be investigated. Third, the enrichment of the proposed control scheme with appropriately designed observers is expected to contribute to a satisfactory solution of the diagnosis problem.

## Figures and Tables

**Table 1:** Simulation of the wafer manufacturing process for a sequence of events.

Event	$\mathrm{Robot}\;R_{1}$	$\mathrm{Robot}\;R_{2}$	$\mathrm{Station}\;C_{1,1}$	$\mathrm{Station}\;C_{2,1}$	$\mathrm{Buffer}\;B_{1}$	$\mathrm{Buffer}\;B_{2}$
-	q1R,1	q2R,1	q1,11	q2,11	q11	q2,11
$e_{I}$	q1R,2	q2R,1	q1,11	q2,11	q11	q2,11
e1,1D	q1R,1	q2R,1	q1,12	q2,11	q11	q2,11
e1,1P	q1R,1	q2R,1	q1,12	q2,11	q11	q2,11
e1,1u	q1R,1	q2R,1	q1,13	q2,11	q11	q2,11
e1,1P	q1R,2	q2R,1	q1,11	q2,11	q11	q2,11
e1,1D	q1R,2	q2R,1	q1,11	q2,11	q11	q2,11
e1,1BD	q1R,1	q2R,1	q1,11	q2,11	q12	q2,11
$e_{I}$	q1R,2	q2R,1	q1,11	q2,11	q12	q2,11
e2,1BP	q1R,2	q2R,2	q1,11	q2,11	q11	q2,11
e1,1D	q1R,1	q2R,2	q1,12	q2,11	q11	q2,11
e2,1D	q1R,1	q2R,1	q1,12	q2,12	q11	q2,11
e1,1u	q1R,1	q2R,1	q1,11	q2,12	q11	q2,11
e1,1P	q1R,1	q2R,1	q1,13	q2,12	q11	q2,11
e2,1u	q1R,1	q2R,1	q1,13	q2,13	q11	q2,11
e2,1P	q1R,1	q2R,2	q1,13	q2,11	q11	q2,11
e1,1P	q1R,2	q2R,2	q1,13	q2,11	q11	q2,11
e2,1BD	q1R,2	q2R,1	q1,13	q2,11	q11	q2,12
e1,1BD	q1R,1	q2R,1	q1,13	q2,11	q12	q2,12

## Data Availability

Data is contained within the article and Supplementary Material.

## Supplementary Materials

The following supporting information can be downloaded at: https://www.mdpi.com/article/10.3390/s23094545/s1, Table S1: List of Acronyms; Table S2: List of symbols.

## Appendix A

**Proof of Theorem** **3.** Any state of G˜ic is of the following 12-tuple form:

(qiR,qi,1,qi,2,qi,qi−1,qiR,S,qi,1S,qi,2S,qiS,qiB.S,qi,1D,S,qi,2D,S)∈      ℚiR×ℚi,1×ℚi,2×ℚim×ℚi−1m×ℚiR,S×ℚi,1S×ℚi,2S×ℚiS×ℚiB.S×ℚi,1D,S×ℚi,2D,S.

It is important to mention that all states of the supervisors in (49a) are marked states. Regarding the automata Gi,1c, Gi,2c, G˜i, and G˜i−1, it is noted that according to Lemma 1 and Lemma 2, all of their states are marked. Thus, the characteristic of a state of G˜ic to be or not be a marked state depends entirely upon the respective element of the 12-tuple state, being a state of GiR. Hence, for G˜ic to be a nonblocking automaton, it suffices to prove that

ℍic(qiR,qi,1,qi,2,qi,qiR,S,qi,1S,qi,2S,qiS,qi+1S,qiB.S,qi,1D,S,qi,2D,S)∩ℍiR(qiR,2)≠∅

where ℍic(·) is the active event set per state of G˜ic, and ℍiR(qiR,2)={ei,1D,ei,2D,ei,i−1BD,ei,iBD}. In other words, it suffices to prove that when the robotic manipulator picks a product, i.e., the automaton GiR is in the state qiR,2, then a drop event is always in the active event set of the state of G˜ic, having as an element the state qiR,2, to guarantee that the robotic manipulator has the ability to drop the product, and consequently the automaton GiR is able to return to the marked state qiR,1. The alphabet of SiR is EiR. Hence, it holds that GiR||SiR=GiR×SiR, where “×” denotes the product of two automata (see [16]). Thus, according to the product definition, it holds that ℍiR||S(qiR,qiR,S)=ℍiR(qiR)∩ℍiR,S(qiR,S), where ℍiR||S is the active event set of the automaton GiR||SiR. Since ℍiR,S(qiR,S)⊆ℍiR(qiR), it is observed that the following equality holds true: ℍiR||S(qiR,qiR,S)=ℍiR,S(qiR,S). The transition of GiR from qiR,1 to qiR,2 can be accomplished only through a pick event, i.e., an event of the set {ei,1P,ei,2P,ei,i−1BP,ei,iBP}. In what follows, the restrictions of the supervisors and the automata Gi,1c, Gi,2c, G˜i, and G˜i−1 to all possible transitions from qiR,2 to qiR,1 will be investigated by considering four different cases, for the arrival from qiR,1 to qiR,2. Taking into account the accessible states of the synchronous product in (49a), it is observed that if GiR is in its initial state qiR,1, then SiR is in its initial state qiR,S,1. Thus, the transitions from qiR,1 to qiR,2 through a pick event corresponds to the transition of SiR from qiR,S,1 to the respective one of the supervisor’s rest four states. Based on this property, the four cases will be presented. *Case 1*: The transition from the initial state qiR,1 to qiR,2, through the event ei,1P is investigated. Regarding GiR||SiR, it is observed that if the event ei,1P takes place, then GiR arrives at qiR,2 and SiR arrives at qiR,S,3. Note that ℍiR,S(qiR,S,3)={ei,iBD}; thus, it holds that ℍiR||S(qiR,2,qiR,S,3)={ei,iBD}. Regarding the rest supervisors, i.e., the supervisors SiB, Si,1D, and Si,2D, it is observed that their alphabets do not contain the event ei,iBD. Regarding the automata Gi,1c, Gi,2c, and G˜i−1, it is observed that their alphabets do not contain the event ei,iBD. Thus, these supervisors and the automata Gi,1c, Gi,2c, and G˜i−1 do not restrict the transitions of the synchronous product in (49a) through the event ei,iBD. From Figure 13, it is observed that if Si is in qiS,1, then if the event ei,1P takes place, then the supervisor Si arrives at qiS,2. Clearly, it holds that ℍiS(qiS,2)={ei,iBD}. Regarding G˜i, it is mentioned that its alphabet contains the event ei,iBD. From Figure 3, it is observed that if G˜i is in qi1, then if the event ei,1P takes place, then G˜i stays in qi1. Clearly, it holds that ℍi(qi1)={ei,iBD}. Regarding GiR||G˜i||SiR||Si, it holds that

ℍicqiR,2,qiR,S,3,qi1,qiS,2=ℍiR||S(qiR,2,qiR,S,3)∩ℍi(qi1)∩ℍiS(qiS,2)={ei,iBD}

Thus, the transition of GiR from qiR,2 to qiR,1 is feasible through ei,iBD. If G˜i or Si are not in qi1 or qiS,1, respectively, i.e., it is in a state where the event ei,1P is not an active event, then no transition of the synchronous product GiR||G˜i||SiR||Si will take place through the event ei,1P, and consequently GiR will be in the marked state qiR,1. *Case 2*: The transition from the initial state qiR,1 to qiR,2 through the event ei,2P is investigated. Regarding GiR||SiR, it is observed that if the event ei,2P takes place, then GiR arrives at qiR,2 and SiR arrives at qiR,S,5. Note that ℍiR,S(qiR,S,5)={ei,i−1BD}; thus, it holds that ℍiR||S(qiR,2,qiR,S,5)={ei,i−1BD}. Regarding the rest supervisors, i.e., the supervisors Si, Si,1D, and Si,2D, it is observed that their alphabets do not contain the event ei,i−1BD. Regarding the automata Gi,1c, Gi,2c, and G˜i, it is observed that their alphabets do not contain the event ei,iBD. Thus, these supervisors and the automata Gi,1c, Gi,2c, and G˜i do not restrict the transitions of the synchronous product in (49a) through the event ei,i−1BD. From Figure 13, it is observed that if SiB is in qiS,B,1, then if the event ei,i−1BD takes place, then the supervisor SiB arrives at qiB,S,4. Clearly, it holds that ℍiB,S(qiB,S,4)={ei,i−1BD}. Regarding G˜i−1, it is mentioned that its alphabet contains the event ei,i−1BD. From Figure 3, it is observed that if G˜i−1 is in qi1, then if the event ei,2P takes place, then G˜i−1 stays in qi1. Clearly, it holds that ℍi−1(qi−11)={ei,i−1BD}. Regarding GiR||G˜i−1||SiR||SiB, it holds that

ℍiGiR||G˜i−1||SiR||SiBqiR,2,qiR,S,5,qi−11,qiB,S,4=ℍiR||S(qiR,2,qiR,S,5)∩ℍi−1(qi−11)∩ℍiB(qi1,qiB,S,4)={ei,i−1BD}

where ℍiGiR||G˜i−1||SiR||SiB·,·,·,· denotes the active event sets of the states of GiR||G˜i−1||SiR||SiB. Thus, the transition of GiR from qiR,2 to qiR,1, under the influence of G˜i−1, SiR and SiB, is feasible through ei,i−1BD. If G˜i−1 is not in qi−11 or SiB is not in qiB,S,4, the automaton GiR||G˜i−1||SiR||SiB is in a state where the event ei,2P is not an active event and no transition of GiR||G˜i−1||SiR||SiB will take place, through the event ei,2P. Consequently GiR will be in the marked state qiR,1. *Case 3*: The transition from the initial state qiR,1 to qiR,2 through the event ei,i−1BP is investigated. Regarding GiR||SiR, it is observed that if the event ei,i−1BP takes place, then GiR arrives at qiR,2 and SiR arrives at qiR,S,2. Note that ℍiR,S(qiR,S,2)={ei,1D}; thus, it holds that ℍiR||S(qiR,2,qiR,S,2)={ei,1D}. Regarding the rest supervisors, i.e., the supervisors Si, SiB, Si,1D, and Si,2D, it is observed that their alphabets do not contain the event ei,1D. Regarding the automata Gi,2c, G˜i, and G˜i−1, it is observed that their alphabets do not contain the event ei,1D. Thus, these supervisors and the automata Gi,2c, G˜i, and G˜i−1 do not restrict the transitions of the synchronous product in (49a) through the event ei,1D. Regarding Gi,1c, it is mentioned that its alphabet contains the event ei,1D. From Figure 2, it is observed that if Gi,1c is in (qi,11,qi,1S,1), then if the event ei,i−1BP takes place, then Gi,1c stays at (qi,11,qi,1S,1). Clearly, it holds that ℍi,1c(qi,11,qi,1S,1)={ei,1D}. Regarding GiR||Gi,1c||SiR, it holds that

ℍicqiR,2,qiR,S,2,(qi,11,qi,1S,1)=ℍiR||S(qiR,2,qiR,S,2)∩ℍi,1c(qi,11,qi,1S,1)={ei,1D}

Thus, the transition of GiR from qiR,2 to qiR,1 is feasible through ei,1D. If Gi,1c is not in (qi,11,qi,1S,1), i.e., it is in a state where the event ei,i−1BP is not an active event, then no transition of the synchronous product GiR||Gi,1c||SiR will take place through the event ei,i−1BP, and consequently GiR will be in the marked state qiR,1. *Case 4*: the transition from the initial state qiR,1 to qiR,2 through the event ei,iBP is investigated. Regarding GiR||SiR, it is observed that if the event ei,iBP takes place, then GiR arrives at qiR,2 and SiR arrives at qiR,S,4. Note that ℍiR,S(qiR,S,4)={ei,2D}; thus, it holds that ℍiR||S(qiR,2,qiR,S,4)={ei,2D}. Regarding the rest supervisors, i.e., the supervisors Si, SiB, Si,1D, and Si,2D, it is observed that their alphabets do not contain the event ei,2D. Regarding the automata Gi,1c, G˜i, and G˜i−1, it is observed that their alphabets do not contain the event ei,2D. Thus, these supervisors and the automata Gi,1c, G˜i, and G˜i−1 do not restrict the transitions of the synchronous product in (49a) through the event ei,2D. Regarding Gi,2c, it is mentioned that its alphabet contains the event ei,2D. From Figure 2, it is observed that if Gi,2c is in (qi,21,qi,2S,1), then if the event ei,iBP takes place, then Gi,2c stays at (qi,21,qi,2S,1). Clearly, it holds that ℍi,2c(qi,21,qi,2S,1)={ei,2D}. Regarding GiR||Gi,2c||SiR, it holds that

ℍicqiR,2,qiR,S,4,(qi,21,qi,2S,1)=ℍiR||S(qiR,2,qiR,S,4)∩ℍi,2c(qi,21,qi,2S,1)={ei,2D}

Thus, the transition of GiR from qiR,2 to qiR,1 is feasible through ei,2D. If Gi,2c is not in (qi,21,qi,2S,1), respectively, i.e., it is in a state where the event ei,iBP is not an active event, then no transition of the synchronous product GiR||Gi,2c||SiR will take place through the event ei,iBP, and consequently GiR will be in the marked state qiR,1. From the above four cases, it is concluded that if a pick event takes place, then if the transition through a pick event is allowable by the synchronous product in (49a), then the automaton GiR will arrive at the non-marked state qiR,2. The return from qiR,2 to qiR,1 is always allowable through a drop event. Hence, G˜ic is a nonblocking automaton. □

## Appendix B

**Proof of Theorem** **4.** Any state of G˜n+1c is of the following 2m+7-tuple form:

(qn+1R,(qn+1,1,qn+1,1S),…,(qn+1,m,qn+1,mS),qn,qn+1R,S,qn+1B,S,qn+1,2D,S)∈             ℚn+1R×(ℚn+1,1×ℚn+1,1S)×⋯×(ℚn+1,m×ℚn+1,mS)×ℚnm×ℚn+1R,S×ℚn+1B,S×ℚn+1,2D,S

It is important to mention that all states of the supervisors are marked states. According to Lemma 1 and Lemma 2, it is mentioned that all states of the automata Gn+1,1c to Gn+1,mc and G˜n are marked. Thus, the characteristic of a state of G˜n+1c to be or not be a marked state depends entirely upon the state of Gn+1R. Hence, for G˜n+1c to be a nonblocking automaton, it suffices to prove that

ℍn+1cqn+1R,(qn+1,1,qn+1,1S),…,(qn+1,m,qn+1,mS),qn,qn+1R,S,qn+1B,S,qn+1,2D,S∩ℍn+1R(qn+1R,2)≠∅

where ℍn+1c(·) is the active event set per state of G˜n+1c, and ℍn+1R(qn+1R,2)={en+1,1D,en+1,2D,…,en+1,mD,en+1,nBD}. In other words, it suffices to prove that when the robotic manipulator picks a product, i.e., the automaton Gn+1R is in the state qn+1R,2, then a drop event is always in the active event set of the respective state of G˜n+1c, thus allowing the robotic manipulator to drop the product and consequently the automaton Gn+1R to return to the marked state qiR,1. The alphabet of Sn+1R is En+1R. Thus, ℍn+1R||S(qn+1R,qn+1R,S)=ℍn+1R(qn+1R)∩ℍn+1R,S(qn+1R,S), where ℍn+1R||S is the active event set per state of Gn+1R||Sn+1R. Since ℍn+1R,S(qn+1R,S)⊆ℍn+1R(qn+1R), it is observed that ℍn+1R||S(qn+1R,qn+1R,S)=ℍn+1R,S(qn+1R,S). The transition of Gn+1R from qn+1R,1 to qn+1R,2 can be accomplished only through a pick event, i.e., an event of the set {en+1,nBP,en+1,1P,en+1,2P,…,en+1,mP}. In what follows, the restrictions of the supervisors in (49b) to all possible transitions from qn+1R,2 to qn+1R,1 in the synchronous product (49b) will be investigated by considering all possible cases for the arrival from qn+1R,1 to qn+1R,2. The number of the cases is five. Taking into account the accessible states of the synchronous product in (49b), it is observed that if Gn+1R is in its initial state qn+1R,1, then Sn+1R is also in its initial state qn+1R,S,1. Thus, the transition from qn+1R,1 to qn+1R,2, through a pick event, corresponds to the transition of Sn+1R from qn+1R,S,1 to one of each m other states. *Case 1*: The transition from the initial state qn+1R,1 to qn+1R,2 through the event en+1,1P is investigated. Regarding Gn+1R||Sn+1R, it is observed that if the event en+1,1P takes place, then Gn+1R arrives at qn+1R,2 and Sn+1R arrives at qn+1R,S,3. Note that ℍn+1R,S(qn+1R,S,3)={en+1,3D}; thus, it holds that ℍn+1R||S(qn+1R,2,qn+1R,S,3)={en+1,3D}. Regarding the supervisors Sn+1B and Sn+1,2D, it is observed that their alphabets do not contain the event en+1,3D. Regarding the automata Gn+1,1c, Gn+1,3c to Gn+1,mc, and G˜n, it is observed that their alphabets do not contain the event en+1,3D. Thus, the supervisor Sn+1B and Sn+1,2D, as well as the automata Gn+1,1c, …, Gn+1,mc, and G˜n, do not restrict the transitions of the synchronous product in (49b) through the event en+1,3D. Regarding Gn+1,2c, it is observed that its alphabet contains the event en+1,3D. From Figure 2, it is observed that if Gn+1,2c is in (qn+1,21,qn+1,2S,1) and the event en+1,1P takes place, then Gn+1,2c stays at (qn+1,21,qn+1,2S,1). Clearly, it holds that ℍn+1,2c(qn+1,21,qn+1,2S,1)={en+1,3D}. Regarding Gn+1R||Sn+1R||Gn+1,2c, it holds that

ℍn+1cqn+1R,2,qn+1R,S,3,(qn+1,21,qn+1,2S,1)=ℍn+1R||S(qn+1R,2,qn+1R,S,3)∩ℍn+1,2c(qn+1,21,qn+1,2S,1)={en+1,3D}

Hence, the transition of Gn+1R from qn+1R,2 to qn+1R,1 is feasible through en+1,3D. If Gn+1,2c is not in (qn+1,21,qn+1,2S,1), then it will be in a state where the event en+1,1P is not an active event and consequently no transition of the synchronous product Gn+1R||Sn+1R||Gn+1,2c will take place through the event en+1,1P. Subsequently the automaton Gn+1R will be in the marked state qn+1R,1. *Case 2*: The transition from the initial state qn+1R,1 to qn+1R,2 through the event en+1,2P is investigated. Regarding Gn+1R||Sn+1R, it is observed that if the event en+1,2P takes place, then Gn+1R arrives at qn+1R,2 and Sn+1R arrives at qn+1R,S,m+2. Note that ℍn+1R,S(qn+1R,S,m+2)={en+1,nBD}; thus, ℍn+1R||S(qn+1R,2,qn+1R,S,m+2)={en+1,nBD}. Regarding supervisor Sn+1,2D, it is observed that its alphabet does not contain the event en+1,nBD. Regarding the automata Gn+1,1c to Gn+1,mc, it is observed that their alphabets do not contain the event en+1,nBD. Thus, supervisor Sn+1,2D and the automata Gn+1,1c,…., and Gn+1,mc do not restrict the transitions of the synchronous product in (49b) through the event en+1,nBD. From Figure 13, it is observed that if Sn+1B is in qnS,B,1 and the event en+1,2P takes place, then the supervisor Sn+1B arrives at qn+1B,S,2. Clearly, it holds that ℍn+1B,S(qn+1B,S,2)={en+1,nBD}. Regarding G˜n, it is observed that its alphabet contains the event en+1,nBD. From Figure 3, it is observed that if G˜n is in qn1 and the event en+1,2P takes place, then G˜n will stay in qn1. Clearly, it holds that ℍn(qn1)={en+1,nBD}. Regarding Gn+1R||G˜n||Sn+1R||Sn+1B, it holds that

ℍn+1cqn+1R,2,qn+1R,S,m+2,qn1,qn+1B,S,4=ℍn+1R||S(qn+1R,2,qn+1R,S,m+2)∩ℍn(qn1)∩ℍn+1B(qn+1B,S,4)={en+1,nBD}

Hence, the transition of Gn+1R from qn+1R,2 to qn+1R,1 is feasible through en+1,nBD. If G˜n is not in qn1 or Sn+1B is not in qn+1B,S,1, both automata are in states where the event en+1,2P is not active and consequently no transition of the synchronous product Gn+1R||G˜n||Sn+1R||Sn+1B, will take place through en+1,2P. Subsequently Gn+1R will be in the marked state qn+1R,1. *Case 3*: The transition from the initial state qn+1R,1 to qn+1R,2 through en+1,jP, where j∈{3,…,m−1}, is investigated. Regarding Gn+1R||Sn+1R, it is observed that if en+1,jP takes place, then Gn+1R arrives at qn+1R,2 and Sn+1R arrives at qn+1R,S,j+1. Note that ℍn+1R,S(qn+1R,S,j+1)={en+1,j+1D}. Hence, ℍn+1R,R(qn+1R,2,qn+1R,S,j+1)={en+1,j+1D}. Regarding the supervisors Sn+1B and Sn+1,2D, it is observed that their alphabets do not contain the event en+1,j+1D. Regarding the automata Gn+1,1c to Gn+1,j−1c,Gn+1,j+1c to Gn+1,mc, and G˜n, it is observed that their alphabets do not contain the event en+1,j+1D. Thus, the supervisors Sn+1B and Sn+1,2D, as well as the automata Gn+1,1c,…,Gn+1,j−1c,Gn+1,j+1c,…, Gn+1,mc, and G˜n, do not restrict the transitions of the synchronous product in (49b) through the event en+1,j+1D. Regarding Gn+1,jc, it is mentioned that its alphabet contains the event en+1,j+1D. From Figure 2, it is observed that if Gn+1,jc is in (qn+1,j1,qn+1,jS,1) and the event en+1,jP takes place, then Gn+1,jc will stay at (qn+1,j1,qn+1,jS,1). Clearly, it holds that ℍn+1,jc(qn+1,j1,qn+1,jS,1)={en+1,j+1D}. Regarding Gn+1R||Sn+1R||Sn+1,j, it holds that

ℍn+1cqn+1R,2,qn+1R,S,j+1,(qn+1,j1,qn+1,jS,1)=ℍn+1R||S(qn+1R,2,qn+1R,S,j+1)∩ℍn+1,jc(qn+1,j1,qn+1,jS,1)={en+1,j+1D}

Thus, the transition of Gn+1,jc from qn+1R,2 to qn+1R,1 is feasible through en+1,j+1D. If Gn+1,jc is not in (qn+1,j1,qn+1,jS,1), then Gn+1,jc is in a state where the event en+1,jP is not active and no transition of the synchronous product Gn+1R||Sn+1R||Sn+1,j will take place through the event en+1,jP, and consequently Gn+1R will be in the marked state qn+1R,1. *Case 4*: The transition from the initial state qn+1R,1 to qn+1R,2 through the event en+1,mP is investigated. Regarding Gn+1R||Sn+1R, it is observed that if the event en+1,mP takes place, then Gn+1R arrives at qn+1R,2 and Sn+1R arrives at qn+1R,S,m+1. Note that ℍn+1R,S(qn+1R,S,m+1)={en+1,2D}. Hence, ℍn+1R,R(qn+1R,2,qn+1R,S,m+1)={en+1,2D}. Regarding the supervisors Sn+1B and Sn+1,2D, it is observed that their alphabets do not contain the event en+1,2D. Regarding the automata Gn+1,1c,…,Gn+1,m−1c, and G˜n, it is observed that their alphabets do not contain the event en+1,2D. Thus, the supervisors Sn+1B and Sn+1,2D, as well as the automata Gn+1,1c,…,Gn+1,m−1c, and G˜n, do not restrict the transitions of the synchronous product in (49b) through the event en+1,2D. Regarding Gn+1,mc, it is mentioned that its alphabet contains en+1,2D. From Figure 2, it is observed that if Gn+1,mc is in (qn+1,m1,qn+1,mS,1) and the event en+1,mP takes place, then the supervisor Gn+1,mc will stay at (qn+1,m1,qn+1,mS,1). Clearly, it holds that ℍn+1,mc(qn+1,m1,qn+1,mS,1)={en+1,2D}. Regarding Gn+1R||Sn+1R||Gn+1,mc, it holds that

ℍn+1cqn+1R,2,qn+1R,S,m+1,(qn+1,m1,qn+1,mS,1)=ℍn+1R||S(qn+1R,2,qn+1R,S,m+1)∩ℍn+1,mc(qn+1,m1,qn+1,mS,1)={en+1,2D}

Hence, the transition of Gn+1R from qn+1R,2 to qn+1R,1 is feasible through en+1,2D. If Gn+1,mc is not in (qn+1,m1,qn+1,mS,1), then it is in a state where the event en+1,mP is not an active event and consequently no transition of Gn+1R||Sn+1R||Gn+1,mc will take place through the event en+1,mP. Subsequently, Gn+1R will be in the marked state qn+1R,1. *Case 5*: The transition from the initial state qn+1R,1 to qn+1R,2 through en+1,nBP is investigated. Regarding Gn+1R||Sn+1R, it is observed that if en+1,nBP takes place, then Gn+1R arrives at qn+1R,2 and Sn+1R arrives at qn+1R,S,2. Note that ℍn+1R,S(qn+1R,S,2)={en+1,1D}. Hence, ℍn+1R||S(qn+1R,2,qn+1R,S,2)={en+1,1D}. Regarding the supervisors Sn+1B and Sn+1,2D, it is observed that their alphabets do not contain the event en+1,1D. Regarding the automata Gn+1,2c to Gn+1,mc and G˜n, it is observed that their alphabets do not contain the event en+1,1D. Thus, the supervisors Sn+1B and Sn+1,2D, as well as the automata Gn+1,1c to Gn+1,mc and G˜n, do not restrict the transitions of the synchronous product in (49b), through the event en+1,1D. Regarding Gn+1,1c, it is mentioned that its alphabet contains the event en+1,1D. From Figure 2, it is observed that if Gn+1,1c is in (qn+1,11,qn+1,1S,1) and the event en+1,nBP takes place, then Gn+1,1c will stay at (qn+1,11,qn+1,1S,1). Clearly, it holds that ℍn+1,1c(qn+1,11,qn+1,1S,1)={en+1,1D}. Regarding Gn+1R||Sn+1R||Gn+1,1c, it holds that

ℍn+1cqn+1R,2,qn+1R,S,2,(qn+1,11,qn+1,1S,1)=ℍn+1R||S(qn+1R,2,qn+1R,S,2)∩ℍn+1,1c(qn+1,11,qn+1,1S,1)={en+1,1D}

Hence, the transition of Gn+1R from qn+1R,2 to qn+1R,1 is feasible through en+1,1D. If Gn+1,1c is not in (qn+1,11,qn+1,1S,1), then it is in a state where the event en+1,nBP is not an active event and consequently no transition of the synchronous product Gn+1R||Sn+1R||Gn+1,1c will take place through the event en+1,nBP. Subsequently, Gn+1R will be in the marked state qn+1R,1. From the above five cases it is concluded that if a pick event takes place, then if the transition through a pick event is allowable by the synchronous product in (49b), then the automaton Gn+1R will arrive at the non-marked state qn+1R,2. The return from qn+1R,2 to qn+1R,1 is always allowable through a drop event. Hence, G˜n+1c is a nonblocking automaton. □
