# Supplementary material for: A Distributed Supervisor Architecture for a General Wafer Production System
Source: Sensors (Basel). 2023 May 7;23(9):4545. doi: 10.3390/s23094545 (PMC10181753; doi:10.3390/s23094545)
Supplement: Supplementary file 1 [file sensors-23-04545-s001.zip › sensors-2293862-supplementary.pdf]

sensors MDPI

Supplementary Material for

A Distributed Supervisor architecture for a General Wafer Production System

Fotis N. Koumboulis, Dimitrios G. Fragkoulis and Panteleimon Georgakopoulos

In the following table, the acronyms used in the paper are presented.

Table S1. List of Acronyms

IoT	Internet of Things
DES	Discrete Event systems
RW	Ramadge Wonham
PLC	Programmable Logic Controller
RTU	Remote Terminal Units
PR	Physical Realizable
IEC	International Electrotechnical Commission
SCADA	Supervisory Control and Data Acquisition
MES	Manufacturing Execution System
OPC UA	Open Platform Communications Unified Architecture
LD	Ladder Diagram

In the following table, the list of symbols, used in the paper, is presented.

Table S2. List of symbols.

$n+1$	The number of robotic manipulators of the system.
m	The number of production stations served by the $n+1$-th (last) robotic manipulator.
R_i	The physical entity of the i-th robotic manipulator, where i is an integer between 1 and $n+1$.
$C_{i,j}$	The physical entity of the (i,j) production station, being served by R_i, where j is an integer between 1 and 2, for the first n robots, and an integer between 1 and m, for the last robot.
B_v	The physical entity of the v-th buffer, being served by R_v, where v is an integer between 1 and n.
L_{in}, L_{out}	The physical entities of the input and output loading docks, respectively.
U_i	The physical entity of the control device controlling R_i, where i is an integer between 1 and $n+1$.
$\mathbb{J}(i)$	The set of the production stations indices server by R_i.
${}^i\mathbf{G}_R$, ${}^{i,j}\mathbf{G}$, ${}^v\mathbf{G}$	The mathematical entities (automata) modelling R_i, $C_{i,j}$ and B_v, respectively.
${}^i\mathbb{Q}_R$, ${}^{i,j}\mathbb{Q}$, ${}^v\mathbb{Q}$	The set of states of ${}^i\mathbf{G}_R$, ${}^{i,j}\mathbf{G}$, ${}^v\mathbf{G}$, respectively.
${}^iq_{R,k}$, ${}^{i,j}q_k$, ${}^vq_k$	The k-th state of ${}^i\mathbf{G}_R$, ${}^{i,j}\mathbf{G}$, ${}^v\mathbf{G}$, respectively, where $k \in \mathbb{Z}_+$.
${}^i\mathbb{E}_R$, ${}^{i,j}\mathbb{E}$, ${}^v\mathbb{E}$	The alphabets of ${}^i\mathbf{G}_R$, ${}^{i,j}\mathbf{G}$, ${}^v\mathbf{G}$, respectively.

${}^{i}\mathbb{E}_{R,c}$, ${}^{i}\mathbb{E}_{R,uc}$, ${}^{i,j}\mathbb{E}_{c}$, ${}^{i,j}\mathbb{E}_{uc}$, ${}^{v}\mathbb{E}_{c}$, ${}^{v}\mathbb{E}_{uc}$	The controllable and uncontrollable events sets of ${}^{i}\mathbf{G}_R$, ${}^{i,j}\mathbf{G}$, ${}^{v}\mathbf{G}$, respectively.
${}^{i,j}e_{\phi}$	The event of ${}^{i}\mathbf{G}_R$, ${}^{i,j}\mathbf{G}$, ${}^{v}\mathbf{G}$, where $\varphi \in \{P, D, BP, BD, u\}$, P is for Pick, D is for Drop, BP is for Buffer Pick, BD is for Buffer Drop, and u is for the uncontrollable events.
${}^{i}\mathbb{H}_R(\bullet)$, ${}^{i,j}\mathbb{H}(\bullet)$, ${}^{v}\mathbb{H}(\bullet)$	The active event sets of the argument states of ${}^{i}\mathbf{G}_R$, ${}^{i,j}\mathbf{G}$, ${}^{v}\mathbf{G}$, respectively.
${}^{i}f_R(\bullet,\bullet)$, ${}^{i,j}f(\bullet,\bullet)$, ${}^{v}f(\bullet,\bullet)$	The transition function of the argument states and events ${}^{i}\mathbf{G}_R$, ${}^{i,j}\mathbf{G}$, ${}^{v}\mathbf{G}$, respectively.
${}^{i}x_{R,0}$, ${}^{i,j}x_0$, ${}^{v}x_0$	The initial state of ${}^{i}\mathbf{G}_R$, ${}^{i,j}\mathbf{G}$, ${}^{v}\mathbf{G}$.
${}^{i}\mathbb{Q}_{R,m}$, ${}^{i,j}\mathbb{Q}_m$, ${}^{v}\mathbb{Q}_m$	The set of the marked states of ${}^{i}\mathbf{G}_R$, ${}^{i,j}\mathbf{G}$, ${}^{v}\mathbf{G}$, respectively.
$\mathbb{L}(\bullet)$, $\mathbb{L}_m(\bullet)$	The closed and the marked behavior of the argument automaton.
$\mathbf{G}_I$, $\mathbf{G}_O$	The mathematical entities (automata) modelling L_{in} and L_{out}.
$\mathbb{Q}_I$, $\mathbb{Q}_O$	The set of states of $\mathbf{G}_I$, $\mathbf{G}_O$, respectively.
$q_{I,1}$, $q_{O,1}$	The state of $\mathbf{G}_I$, $\mathbf{G}_O$, respectively.
$\mathbb{E}_I$, $\mathbb{E}_{I,c}$, $\mathbb{E}_{I,uc}$, $\mathbb{E}_O$, $\mathbb{E}_{O,c}$, $\mathbb{E}_{O,uc}$,	The alphabets, the controllable and the uncontrollable events sets of $\mathbf{G}_I$, $\mathbf{G}_O$, respectively.
e_I, e_O	The event of $\mathbf{G}_I$, $\mathbf{G}_O$, respectively.
$\mathbb{H}_I(\bullet)$, $\mathbb{H}_O(\bullet)$	The active event sets of the argument states of $\mathbf{G}_I$, $\mathbf{G}_O$, respectively.
$f_I(\bullet,\bullet)$, $f_O(\bullet,\bullet)$	The transition functions of the argument states and events of $\mathbf{G}_I$, $\mathbf{G}_O$, respectively.
$x_{I,0}$, $\mathbb{Q}_I$, $x_{O,0}$, $\mathbb{Q}_O$	The initial state and the set of the marked states of $\mathbf{G}_I$, $\mathbf{G}_O$, respectively.
${}^{i,j}\mathbb{K}$, ${}^{i}\mathbb{K}$, ${}^{i}\mathbb{K}_B$, ${}^{i}\mathbb{K}_R$, ${}^{i,j}\mathbb{K}_D$	The desired regular languages related to the influence of U_i.
${}^{i,j}\tilde{\mathbb{E}}$, ${}^{i}\tilde{\mathbb{E}}_R$, ${}^{i}\tilde{\mathbb{E}}$, ${}^{i}\tilde{\mathbb{E}}_B$, ${}^{i,j}\tilde{\mathbb{E}}_D$	The alphabets of ${}^{i,j}\mathbb{K}$, ${}^{i}\mathbb{K}$, ${}^{i}\mathbb{K}_B$, ${}^{i}\mathbb{K}_R$, ${}^{i,j}\mathbb{K}_D$, respectively.
${}^{i,j}P$, ${}^{i,j}\tilde{P}$, ${}^{i,j}P_D$	The projections of ${}^{i}\tilde{\mathbb{E}}_R^*$ to ${}^{i,j}\mathbb{E}^*$, ${}^{i,j}\tilde{\mathbb{E}}^*$, ${}^{i,j}\tilde{\mathbb{E}}_D^*$, respectively.
${}^{i}P$, ${}^{i}\tilde{P}$, ${}^{i}P_B$, ${}^{i}\tilde{P}_B$, ${}^{i}P_R$	The projections of ${}^{i}\tilde{\mathbb{E}}_R^*$ to ${}^{i}\mathbb{E}^*$, ${}^{i}\tilde{\mathbb{E}}^*$, ${}^{i-1}\tilde{\mathbb{E}}^*$, ${}^{i}\tilde{\mathbb{E}}_B^*$, ${}^{i}\mathbb{E}_R^*$, respectively.
${}^{i,j}\mathbf{S}$, ${}^{i}\mathbf{S}$, ${}^{i}\mathbf{S}_B$, ${}^{i}\mathbf{S}_R$, ${}^{i,j}\mathbf{S}_D$	The supervisor automata realizing ${}^{i,j}\mathbb{K}$, ${}^{i}\mathbb{K}$, ${}^{i}\mathbb{K}_B$, ${}^{i}\mathbb{K}_R$, ${}^{i,j}\mathbb{K}_D$, respectively.
${}^{i,j}\mathbb{Q}_S$, ${}^{i}\mathbb{Q}_S$, ${}^{i}\mathbb{Q}_{R,S}$, ${}^{i,j}\mathbb{Q}_{D,S}$	The set of states of ${}^{i,j}\mathbf{S}$, ${}^{i}\mathbf{S}$, ${}^{i}\mathbf{S}_R$, ${}^{i,j}\mathbf{S}_D$, respectively.
${}^{i,j}q_{S,k}$, ${}^{i}q_{S,k}$, ${}^{i}q_{R,S,k}$, ${}^{i,j}q_{D,S,k}$	The k-th state of ${}^{i,j}\mathbf{S}$, ${}^{i}\mathbf{S}$, ${}^{i}\mathbf{S}_R$, ${}^{i,j}\mathbf{S}_D$, respectively.
${}^{i,j}\mathbb{H}_S(\bullet)$, ${}^{i}\mathbb{H}_S(\bullet)$, ${}^{i}\mathbb{H}_{R,S}(\bullet)$, ${}^{i,j}\mathbb{H}_{D,S}(\bullet)$.	The active event sets of the argument states of ${}^{i,j}\mathbf{S}$, ${}^{i}\mathbf{S}$, ${}^{i}\mathbf{S}_R$, ${}^{i,j}\mathbf{S}_D$, respectively.
${}^{i,j}f_S(\bullet,\bullet)$, ${}^{i}f_S(\bullet,\bullet)$, ${}^{i}f_{R,S}(\bullet,\bullet)$, ${}^{i,j}f_{D,S}(\bullet,\bullet)$	The transition functions of the argument states and events of ${}^{i,j}\mathbf{S}$, ${}^{i}\mathbf{S}$, ${}^{i}\mathbf{S}_R$, ${}^{i,j}\mathbf{S}_D$, respectively.

${}^{i,j}x_{S,0}$, ${}^{i}x_{S,0}$, ${}^{i}x_{R,S,0}$, ${}^{i,j}x_{D,S,0}$,	The initial state of ${}^{i,j}\mathbf{S}$, ${}^{i}\mathbf{S}$, ${}^{i}\mathbf{S}_R$, ${}^{i,j}\mathbf{S}_D$, respectively.
${}^{i,j}\mathbb{Q}_{S,m}$, ${}^{i}\mathbb{Q}_{S,m}$, ${}^{i}\mathbb{Q}_{R,S,m}$, ${}^{i,j}\mathbb{Q}_{D,S,m}$	The set of the marked states of ${}^{i,j}\mathbf{S}$, ${}^{i}\mathbf{S}$, ${}^{i}\mathbf{S}_R$, ${}^{i,j}\mathbf{S}_D$, respectively.
